# Study on Floral Syndrome and Breeding System of an Endangered Species *Rhododendron nymphaeoides*

**DOI:** 10.1002/ece3.72812

**Published:** 2025-12-22

**Authors:** Jun Luo, Meng Chen, Haiyan Long, Yuting Zhu, Congjun Yuan, Kai Hu, Jin Chen, Run Liu, Xiaoyong Dai, Fangjun Ding

**Affiliations:** ^1^ College of Forestry Guizhou University Guiyang Guizhou China; ^2^ Guizhou Academy of Forestry Guiyang Guizhou China; ^3^ Key Laboratory of National Forestry and Grassland Administration on Biodiversity Conservation in Karst Mountainous Areas of Southwestern China Guiyang Guizhou China; ^4^ Guizhou Libo Karst Forest Ecosystem National Observation and Research Station Libo Guizhou China; ^5^ Guizhou Technological College of Ecology and Energy Guiyang Guizhou China

**Keywords:** breeding system, flowering phenology, flower‐visiting insects, pollen characteristics, pollination biology, *Rhododendron nymphaeoides*

## Abstract

*Rhododendron nymphaeoides* is a plant belonging to the genus *Rhododendron* (*Rhododendron* Linn.) and the subsection *Fortunea*. Although this species is clearly listed as endangered (with an endangered status of EN) by the International Union for Conservation of Nature (IUCN) Red List, the Red List of Rhododendrons, the Red List of China's Higher Plants, and the Threatened Species List of China's Higher Plants, the question of whether its mating system is self‐pollinating or cross‐pollinating remains a perplexing issue that hinders the conservation and management of wild resources. This study investigated the flowering phenology, floral characteristics, pollen traits, breeding system, and pollination biology of *R. nymphaeoides*. The results indicate that (1) the peak flowering period for *R. nymphaeoides* occurs in late April, with the flowering process of a single flower divided into five stages: bud stage, bud swelling stage, initial flowering stage, peak flowering stage, and wilting stage, exhibiting synchronous flowering; (2) the pollen of *R. nymphaeoides* is tetrahedral, arranged in a regular tetrahedral formation, with an average diameter of 56.47 μm. The individual pollen grains are nearly spherical with a surface covered in sticky threads, featuring three pores with indistinct ornamentation around the grooves, and the outer wall consists of two layers with a faint reticulate sculpture visible on the surface; (3) the pollen vitality of *R. nymphaeoides* is relatively strong throughout the flowering period, peaking at 11:00 a.m. on the day of flowering at 91.86% and maintaining a 36.67% vitality until the end of the flowering period (9 days after flowering), making it suitable for use as a parent in the hybrid breeding work; (4) the stigma receptivity of *R. nymphaeoides* lasts for a long time, with the optimal pollination period occurring during the peak flowering stage (days 3–6 after flowering); (5) the most suitable culture medium for pollen is 150 g·L^−1^ sucrose + 200 mg·L^−1^ H_3_BO_3_ + 50 mg·L^−1^ CaCl_2_, with the optimal storage temperature being −80°C; (6) the breeding system of *R. nymphaeoides* is classified as a facultative cross‐fertilization mixed mating system, relying on pollinators, with a low natural fruit set rate (43.33%). The fruit set rate for cross‐pollination between different individuals is 83.33%, indicating pollination limitations and weak reproductive assurance (RA); (7) effective pollinators of *R. nymphaeoides* include *Papilio paris*, *Papilio bianor*, *Papilio polytes*, *Papilio protenor*, *Apis cerana* , *Vespula koreensis*, *Vespa velutina*, *Xylocopa appendiculata* , and *Xylocopa tranquebarorum* , while insects such as *Bombus eximius* , *Anthophora plumipes* , *Syrphus torvus* , *Helophilus eristaloidea*, *Eristalis arbustorum,* and *Nemoraea pellucida* act as nectar robbers, and *Osmia taurus* serve as pollen thieves.

## Graphical abstract

This study divided the flowering process of *Rhododendron nymphaeoides* into five periods and systematically investigated the floral traits, pollen morphology and viability, optimal solid media for in vitro pollen germination, storage temperature screening, pollen–ovule ratio and hybridization index, stigma receptivity, the effects of different pollination methods on fruit set rates, and visiting insects. This research aimed to understand the floral syndrome and breeding system of this endangered species.

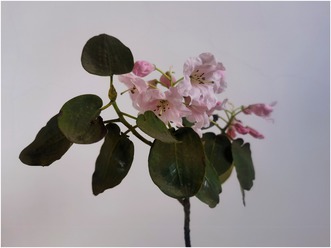

## 1 Introduction

The breeding system of plants has become one of the most active areas of research in ecology and evolutionary biology (Wyatt 1983). It generally refers to the totality of sexual characteristics that influence the genetic composition of offspring, including floral morphological traits, flowering patterns, types of pollinators, self‐compatibility, and mating systems (He and Liu 2003; Bie et al. 2018; Cao et al. 2022). Throughout the long process of evolution, angiosperms have developed a variety of floral syndromes to adapt to unfavorable pollination environments and ensure reproductive success. These include flower design (flower color, flower structure, flower scent, and floral rewards) and flower display (flowering process, flower lifespan, and the number and spatial arrangement of flowers in an inflorescence) (Nicotra et al. 2010). This is a result of reproductive strategies and the joint action of genetic factors under extreme environmental reproductive constraints (Spigler and Kalisz 2013). These characteristics not only affect the attraction to pollinators, pollen dispersal, pollinator visitation behavior, the degree of self‐pollination versus cross‐pollination, and the fitness of male and female plants, but also influence the mating patterns and population dispersal capabilities of plants (Barrett and Harder 1996; D. Y. Zhang 2004; Devaux et al. 2014). Existing research indicates that the breeding system, as a bridge connecting plant sexual reproduction, plays a crucial role not only in determining plant genetic diversity and genetic structure (Loveless and Hamrick 1984) but also in the phenotypic variation and evolutionary pathways of species (Barrett 2010). Therefore, the study of plant breeding systems and their diversification is of great significance for revealing the evolution of various plant groups, exploring species formation, population expansion, and adaptation to the environment (Dai et al. 2013).

A successful conservation program for endangered species must incorporate information on the reproductive biology of the species (Lander et al. 2019). The success of pollination is directly related to whether plants can reproduce sexually (Wu et al. 2022). Pollination is a prerequisite for fruit set; if there are issues during the pollination process, it will affect the fruiting rate and seed set of the plants, which in turn impacts seed germination and seedling establishment, ultimately affecting the natural regeneration of populations and the long‐term survival of the species (Swift et al. 2016). Research by Tai et al. (2023) on the flowering biological characteristics and breeding system of the rare and endangered plant *Sinojackia microcarpa* found that its pollination is easily affected by weather, with few types of pollinating insects and low visitation frequency, leading to very low seed set rates in natural populations. Most seeds are either empty or poorly developed, severely impacting the self‐renewal of the population. Therefore, artificial pollination to increase fruit set and seed set rates, thereby promoting population renewal, is an effective strategy for the conservation of this species. Research by Wan et al. (2018) on the breeding system and pollination biology of the endangered plant *Plantago fengdouensis* indicates that the floral characteristics, flowering phenology, and breeding system of this species provide certain RA to adapt to the flooding stress during the summer in the Three Gorges Reservoir area, ensuring the long‐term survival of the population. The findings can provide theoretical guidance for ex‐situ conservation and in situ protection of this population. It is evident that understanding the reproductive biological characteristics of plants, including pollination syndromes and breeding systems, is helpful in clarifying the reasons for species endangerment, guiding the formulation of appropriate conservation strategies, and carrying out effective rescue and conservation actions (Marbaniang et al. 2018).

**FIGURE 1:**
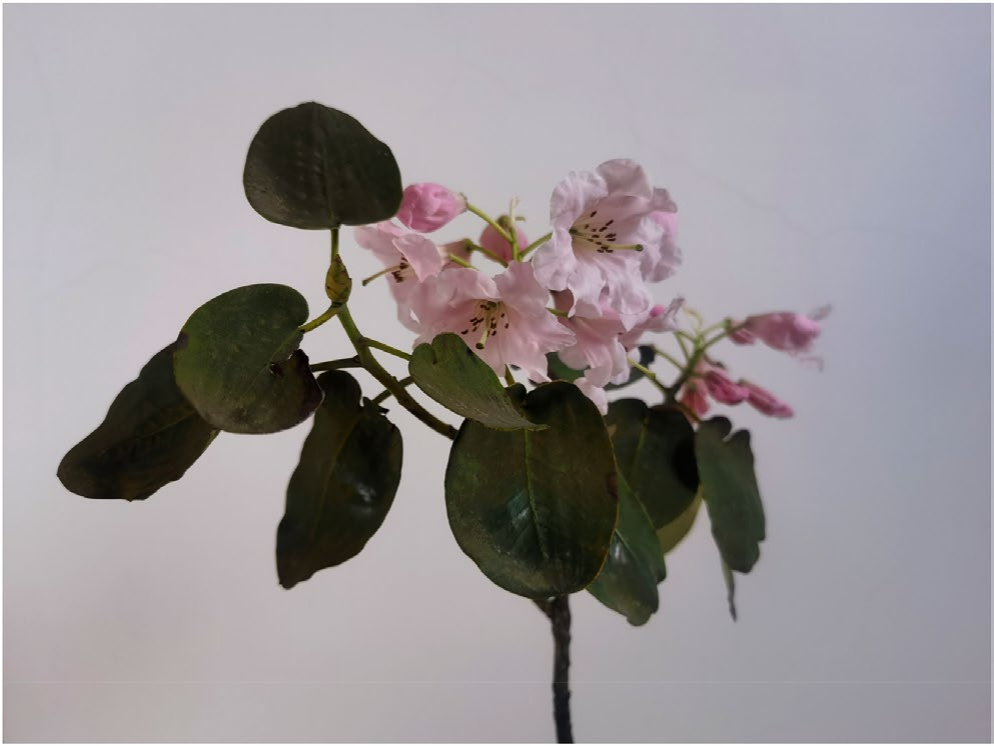
A photograph of *R. nymphaeoides*.

*Rhododendron nymphaeoides* W. K. Hu is a plant belonging to the subgenus *Fortunea* of the genus *Rhododendron*. Its most notable characteristics include ovate leaves with ear‐shaped bases, and the ovary and style are covered with reddish‐brown short‐stalked glands (Figure 1) (Dai et al. 2022). According to the “*Red List of Threatened Species*” published by the International Union for Conservation of Nature (IUCN), the “*Red List of Rhododendrons*” co‐issued by the International Plant Protection Alliance, World Wildlife Fund, Global Trees Campaign, and Royal Botanic Garden Edinburgh, as well as the “*Red List of China's Higher Plants*” published by the Ministry of Ecology and Environment of China, and the “*Threatened Species List of China's Higher Plants*” published by the Chinese Academy of Sciences, *R. nymphaeoides* has been classified as an endangered species. The assessment criteria are B1ab(i,ii,iii), with an endangered status of EN. Its type locality is located in Gulin County, Sichuan Province, China. The habitat destruction is extremely severe, making it one of the 19 *Rhododendron* species globally classified as endangered, necessitating urgent conservation efforts (Luo, Dai, Chen, He, et al. 2025).

In recent years, research on the relationship between the breeding systems of *Rhododendron* species and their environment has received considerable attention. The breeding systems of most *Rhododendron* species, such as *Rhododendron arboreum* (Jain et al. 2000), *Rhododendron ferrugineum* (Escaravage and Wagner 2004), *Rhododendron maxiongense* (Yi et al. 2018), *Rhododendron siderophyllum* (Bai et al. 2014), and *Rhododendron excellens* (Tian et al. 2024), tend to favor a composite mating system that promotes outcrossing. However, studies have also found that the breeding systems of species such as *Rhododendron sinofalconeri* (X. Zhang 2020), *Rhododendron hemsleyanum* (Xie et al. 2023), *Rhododendron griersonianum* (Liu et al. 2025), and *Rhododendron longipedicellatum* (Li et al. 2018) are mixed mating systems that allow for both self‐pollination and cross‐pollination. So, what type of breeding system is attributed to *R. nymphaeoides*? This question has not yet been reported, and it is fundamental to species conservation and population restoration. In light of this, this study takes the type locality of this species, the Hutou Mountain Scenic Area in Gulin County, Sichuan Province, China, as the research site to conduct a detailed study on the flowering phenology, flower syndrome, pollination characteristics, and breeding system of this species, aiming to explore the following questions: (1) What are the characteristics of the flowering phenology of this species? (2) What are the morphological characteristics of its flowers and the flowering process? (3) What type of breeding system is this species classified as? (4) What are the characteristics of its pollen morphology and pollination traits? Through this research, it is hoped that a scientific basis will be provided for the formulation of conservation strategies and artificial propagation for this species.

## 2 Materials and Methods

### 2.1 Overview of the Study Area

**FIGURE 2:**
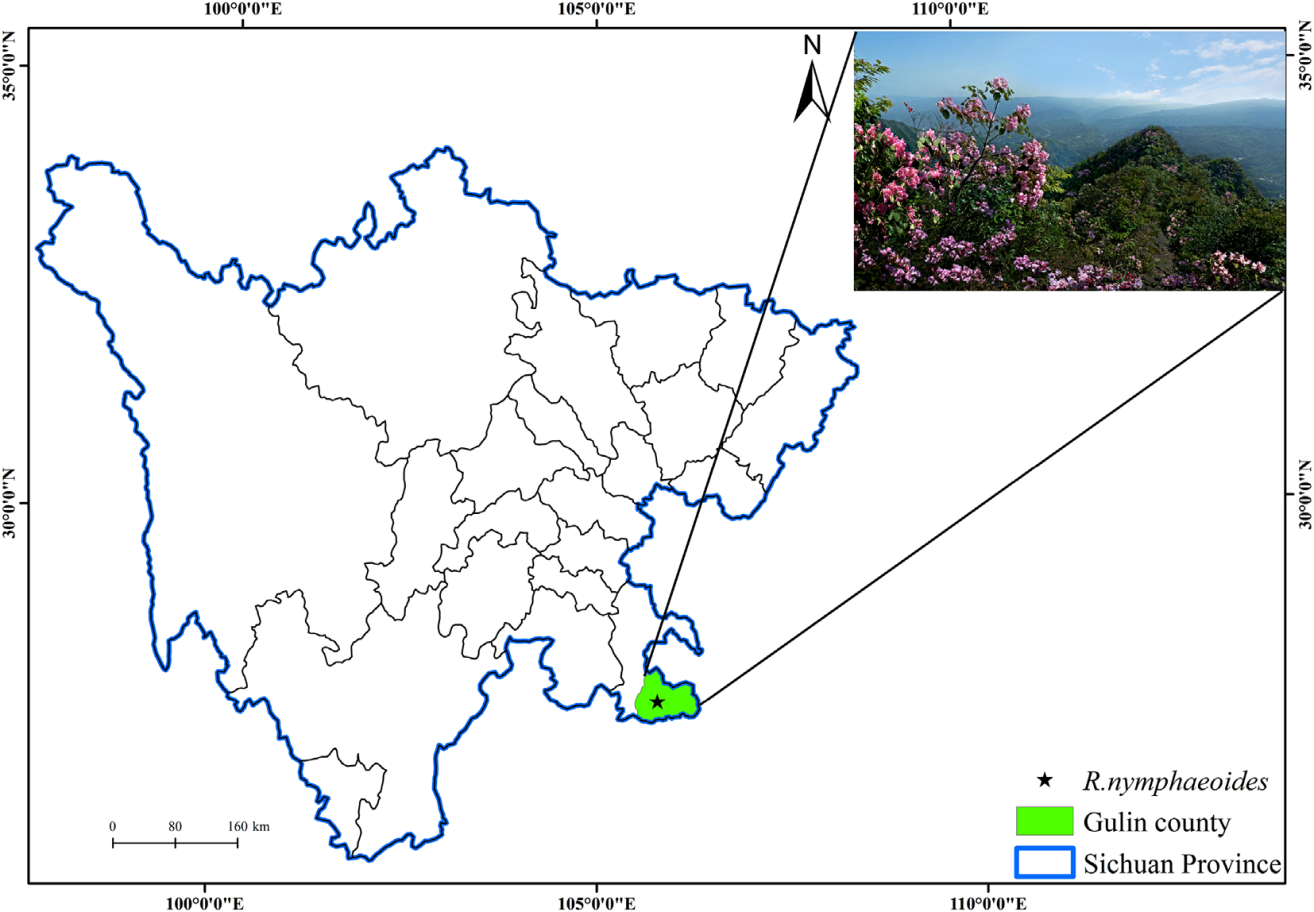
Geographic locations of *R. nymphaeoides* populations sampled in this study.

The research site is located in the Hutoushan Scenic Area of Gulin County, Sichuan Province, China (E 105°45′, N 28°07′, elevation 1707 m) (Figure 2). The Hutoushan Scenic Area in Gulin County is situated on the northern side of the Daluo Mountain Range in the transition zone between the Sichuan Basin and the Yunnan‐Guizhou Plateau, and it is located in the lower reaches of the Chishui River basin. The area has a subtropical monsoon climate, with an average annual temperature ranging from 13.0°C to 18.6°C and annual rainfall between 700 and 800 mm, with purple soil being predominant (Xiao 2010). *R. nymphaeoides* is distributed in the middle and upper parts of the Tiger Head Mountain Scenic Area (at an altitude of 1707.58 m). There are a total of 66 *R. nymphaeoides* plants in this area, among which 47 are mature plants. The associated tree layer primarily consisting of *Osmanthus fragrans* , *Lyonia ovalifolia*, and *Lithocarpus hancei*, while the shrub layer is mainly composed of *Fargesia spathacea* and *Rhododendron simsii* (Luo, Dai, Chen, Yuan, et al. 2025).

### 2.2 Research Materials

Wild *R. nymphaeoides* growing in the study area was surveyed from March to October 2024. The formal identification of the samples used in this study was performed by Xiao‐Yong Dai, a taxonomist of the genus *Rhododendron* spp. Voucher specimens were deposited in the Herbarium of Guizhou Provincial Academy of Forestry, with the deposition number GL20240922‐1. The identification of pollinating insects was completed by Kai Hu, and the voucher specimens were stored in the Insect Specimen Room of the Guizhou Academy of Forestry, with the deposition number IN20240425‐1 to IN20240425‐17. Our field investigations and experimental studies complied with the regulations of local legislative bodies, as well as national and international guidelines.

### 2.3 Observation of Flowering Phenology and Flower Characteristics

From March to May 2024, observations of the flowering phenology of wild populations of *R. nymphaeoides* in the Hutoushan Scenic Area of Gulin County, Sichuan Province, were conducted. According to the principles and methods of reproductive biology, among mature plants, 15 plants were selected based on the principle of a minimum spacing of 5 m per plant to record their initial flowering, final flowering, the duration of the flowering period and the synchronization of flowering. The initial flowering date of an individual was defined as the date when its first flower opened. The flowering peak period for a single plant was identified as the date when 50% of its flowers had opened, and the final flowering date was recorded as the date of the last flower opening. The total flowering duration of an individual was calculated as the period from the first to the last flower opening. Flowering phenological parameters at the individual level were averaged across all marked individuals. For population‐level flowering progression, following Dafni's (1992) method, the initial flowering date was determined as the date when 25% of individuals within the population had initiated flowering. The population flowering peak occurred when 50% of individuals reached their flowering peak, and the final flowering date was defined as the date when 95% of plants had completed flowering. Each plant is randomly marked with three inflorescences. The number of newly opened flowers and the duration of flowering were observed and recorded daily. During the peak flowering period, the morphological characteristics of the flowers of *R. nymphaeoides* were observed and recorded, including petal length, petal width, length of corolla tube, corolla diameter, stigma width, stigma length, pedicel length, filament length, anther length, ovary length, ovary width, and the shortest distance between the stigma and anther, etc. Thirty flowers were randomly tagged, and the flowering duration and morphological changes of individual flowers were recorded, including the first flowering time, last flowering time, and flowering synchrony index (*W*) (Mcintosh 2002). Since this study only investigated the *R. nymphaeoides* population in Hutou Mountain Scenic Area, Gulin County, Sichuan Province, the flowering period synchronization index (*W*) refers to the synchrony among individuals within the same population. The specific formula is:

$$W=\frac{1}{N}\left(\frac{1}{T_{K}}\right)\sum_{J=K}^{N}R_{J\neq K}$$

In this formula, the flowering synchrony index (*W*) ranges from 0 to 1; *N* represents the total number of individuals in the population; *T*_*K*_ is the flowering duration of an individual flower; and *R*_*J*_ is the overlapping flowering time between individual *K* and individual *J*.

The nectar spurs were cut using dissecting scissors, and the nectar volume was measured using a micro‐syringe (5 μL). The sugar content of the nectar was measured using a handheld refractometer (0%–80%), and the peak reflectance spectrum of the petals was measured using an INESA‐L5S spectrophotometer (Basnett et al. 2019; Dellinger 2020).

### 2.4 Observation of Pollen Morphology

The classification of plants is essential to better understand, protect, and utilize plant resources. Pollen characteristics serve as one of the critical bases for plant taxonomy and for exploring plant origin, evolution, and phylogenetic relationships (Li et al. 2018). An in‐depth study of the pollen morphological characteristics of *R. nymphaeoides* can provide crucial evidence for its accurate classification. Therefore, take the 15 *R. nymphaeoides* plants selected in Section 2.3 and the 30 flowers labeled as the objects, fresh pollen at different developmental stages was collected and washed 2–3 times with distilled water to ensure that the pollen grains were fully dispersed. After washing, some pollen grains were placed on a concave microscope slide and observed and photographed under an OLYMPUS‐BX53 microscope. Other pollen grains were placed in a drying dish for dehydration. After drying, they were fixed onto the sample stage using double‐sided conductive tape. Following gold sputter coating, they were observed, photographed, and recorded under a COXEM EM‐30 Plus desktop scanning electron microscope. During the peak flowering period of 30 flower petals, 30 pollen grains were randomly selected to measure their diameter, the length and width of the germination pores, and the relative length of the germination pores. The relative length was calculated as the length of the germination pore divided by the diameter (Li et al. 2018).

### 2.5 Pollen Viability Test

Pollen viability assessment of *R. nymphaeoides* on the day of flowering: Among the 15 *R. nymphaeoides* plants selected in Section 2.3, one single flower was chosen from each plant that bloomed on the same day (i.e., the day the sample bloomed), totaling 15 flowers. Pollen was collected every 2 h from 9:00 to 21:00 and smeared onto a microscope slide. The pollen was stained using a mixture of 5.0% sucrose and 0.5% triphenyl tetrazolium chloride (TTC) and placed in a Petri dish (with moist filter paper inside) to stand for 2 h at 25°C in the dark. The staining results were observed under a microscope, and the staining determination process was repeated three times. The principle of the TTC staining method for identifying pollen viability is that viable pollen has a stronger respiratory action and can reduce colorless TTC to red tetrathiafulvalene (TTF), thereby coloring the pollen itself. The respiration of inactive pollen is relatively weak, and the color change of TTC is not obvious. Therefore, the percentage of stained pollen relative to the total number of observed pollen grains was calculated as the pollen viability value for *R. nymphaeoides* (Hao et al. 2025).

Pollen viability assessment during the flowering period of *R. nymphaeoides*: 30 flowers were marked during the bud stage (mainly referring to 1–2 days before flowering) and from 1 to 9 days after flowering. Every day at 9:00, three individual flowers were randomly selected to measure their pollen viability, and the staining determination process was repeated three times. The measurement method was the same as that used for the pollen viability assessment on the day of flowering (Hao et al. 2025).

### 2.6 Pollen Germination Test

According to the method described by Zhang and Geng (2012), the L_25_(5^3^) orthogonal experiment was utilized to explore the effects of different concentrations of agar (10 g·L^−1^), sucrose (0, 50, 100, 150, 300 g·L^−1^), H3BO3 (0, 50, 100, 200, 300 mg·L^−1^), and CaCl_2_ (0, 50, 150, 250, 350 mg·L^−1^) on the pollen germination of *R. nymphaeoides*, in order to identify suitable solid culture media. The specific method involved taking 15 *R. nymphaeoides* plants selected in Section 2.3 and 30 labeled flowers as the objects, collecting fresh pollen from flowers that opened on the same day from different plants at 9:00 a.m. The pollen was thoroughly mixed and evenly spread onto a double‐well concave microscope slide containing more than 0.1 mL of the culture medium. A cover slip was placed on top, and the slide was placed in a Petri dish lined with three layers of moist filter paper, then incubated in a light culture chamber at 25°C ± 1°C. Observations were conducted every 4 h under a low‐magnification microscope, with appropriate components of the culture medium replenished as needed. Once pollen germination was detected, observations were performed hourly until two consecutive observations revealed nearly identical germination rates and no new pollen grains germinated. This time point was designated as the final termination time. During each observation, 3–5 clear fields of view were selected from the center of the slide (with no fewer than 50 pollen grains in each field) to calculate the germination rate (germination rate = number of germinated pollen grains in a field/total number of pollen grains in that field × 100%). This process was repeated three times. A pollen tube was considered germinated if its length was equal to or exceeded the diameter of the pollen grain, and for tetrads, it was considered germinated if at least one individual pollen grain had germinated.

### 2.7 Determination of Pollen Storage Capacity

Taking 15 *R. nymphaeoides* and 30 labeled flowers selected in Section 2.3 as the objects, on the day of flowering at 9:00 a.m., fresh pollen from 15 different plants was collected and thoroughly mixed, then placed in 1‐mL cryovials with an appropriate amount of desiccant and labeled accordingly. The samples were stored in low‐temperature freezers at room temperature (20°C–25°C), 4°C, −20°C, and −80°C. The pollen stored at room temperature was tested for changes in viability every 2 days, while the pollen stored at 4°C, −20°C, and −80°C was tested every 6 days until the viability dropped below 5%, at which point it could no longer be used for hybridization (Liu et al. 2023). The detection method involved using the most suitable culture medium selected in Section 2.6 to culture the pollen for 17 h and then assessing the germination status, with four repetitions.

### 2.8 Stigma Receptivity Test

Taking the 15 *R. nymphaeoides* selected in Section 2.3 as the objects, every day at 11:00 a.m., two individual flowers at the bud stage and 1–9 days after flowering were randomly selected, for a total of 30 flowers. Following the method described by Dafni (1992), the stigmas were placed on a concave microscope slide, and a reaction solution (V3% hydrogen peroxide: V water: V1% aniline blue = 11:22:4) was added to observe whether the stigmas were stained. The principle of determining the column head reproducibility by the benzidine—hydrogen peroxide method is: The active column head contains active catalase. Under the action of catalase, hydrogen peroxide generates highly oxidizing oxygen atoms that oxidize benzidine, causing it to change color and produce bubbles. After adding this test solution, the active column head will rapidly change color and produce bubbles, while the inactive one will not change color and does not produce bubbles. The strength or weakness of the column head can be judged by the change in color and the number of bubbles. The specific criteria were as follows: if no bubbles or color appeared after the stigma contacted the reaction solution, it was recorded as non‐receptive (0); if 1–6 bubbles formed without a blue reaction, it was recorded as partially receptive (1); if bubbles covered half of the stigma area and a faint blue reaction appeared, it was recorded as receptive (2); if bubbles covered two‐thirds of the stigma area and the blue color deepened, it was recorded as strongly receptive (3); and if bubbles were present on almost the entire stigma with a clear blue reaction, it was recorded as very receptive (4).

### 2.9 Pollen Ovule Ratio and Hybridization Index Calculation

Thirty open flower buds that had not yet dehisced were randomly tagged (three inflorescences collected from each of 10 individual plants). These were brought indoors, and once the anthers dehisced, they were made into an anther suspension for observation and counting of pollen quantity under a compound microscope. The petals, corolla, and stamens of *R. nymphaeoides* were removed, and the ovary was dissected with a scalpel under a dissecting microscope to locate the ovules, which were then counted. According to the standards set by Cruden (1977), the breeding type of *R. nymphaeoides* was determined through the pollen–ovule ratio (P/O), calculated as the number of pollen grains per flower divided by the number of ovules per flower. The classifications were as follows: closed flower fertilization (P/O value = 2.70–5.40); obligate self‐fertilization (P/O value = 18.10–39.00); facultative self‐fertilization (P/O value = 31.90–396.90); facultative cross‐fertilization (P/O value = 244.70–2558.60); and obligate cross‐fertilization (P/O value = 2108.00–19525.00). A smaller pollen–ovule ratio indicates a stronger degree of inbreeding, while a larger ratio indicates a stronger degree of outcrossing.

During the peak flowering period of *R. nymphaeoides*, 30 inflorescences were randomly selected (three inflorescences from each of 10 individual plants) to observe their floral characteristics. Following the criteria established by Dafni and Maués (1998) the hybridization index (OCI) was determined based on three aspects: the corolla diameter (< 1.00 mm = 0; 1.00–2.00 mm = 1; 2.10–6.00 mm = 2; > 6.0 mm = 3), the timing consistency of anther dehiscence and stigma receptivity (synchronous or with the pistil maturing first = 0; with the stamens maturing first = 1), and the spatial relative position of the stigma and anthers (at the same height = 0; at different heights = 1). This sum was used to evaluate the breeding system of *R. nymphaeoides*: closed flower fertilization (OCI = 0); obligate self‐fertilization (OCI = 1); facultative self‐fertilization (OCI = 2); facultative cross‐fertilization (OCI = 3); and obligate cross‐fertilization (OCI ≥ 4).

### 2.10 Pollination Treatment

In April 2024, flowers that were about to bloom were selected, and they were subjected to seven different pollination treatments according to the method described by Tamura and Kudo (2000): (1) Natural pollination: No treatment applied except labeling to monitor pollination and fruit set under natural conditions; (2) direct bagging: Flowers were bagged and labeled to test for autonomous self‐pollination (autogamy); (3) emasculated and bagged: Anthers were removed, flowers were labeled and bagged to test for parthenocarpy; (4) emasculation without bagging: Anthers were removed, flowers were labeled and left open to test for pollinator‐mediated cross‐pollination; (5) artificial self‐pollination: Pollen from the same flower was applied to the stigma, followed by emasculation, labeling, and bagging to assess self‐compatibility; (6) intra‐plant cross‐pollination: Anthers were removed, pollen from other flowers on the same plant was applied, followed by labeling and bagging to evaluate geitonogamous selfing compatibility; (7) inter‐plant cross‐pollination: Anthers were removed, pollen from flowers of different plants was applied, followed by labeling and bagging to test for outcrossing success. The main function of using sulfuric acid paper bags is to isolate foreign pollen and ensure the accuracy of pollination experiments. All treatments were labeled, and the fruit set rate was recorded when the seeds matured. The fruit set rate was calculated as the number of mature fruits divided by the number of treated flowers multiplied by 100%. Additionally, a generalized linear model (GLM) with a binomial distribution and logistic link function was employed to compare the differences in fruit set rates between natural pollination and different strains cross‐pollination treatments.

To investigate whether *R. nymphaeoides* has pollination limitation (pollination limitation, PL), that is, insufficient pollen falling on the head leads to limited fruit setting, the difference between the fruit setting rate of artificial pollination and that of the natural control was taken as the pollination limitation index. If PL < 0, it indicates that there is no pollination limitation. If PL > 0, it indicates that there is a pollination limit. To investigate whether *R. nymphaeoides* exhibits RA, the ratio of fruit set rate in emasculated and non‐bagged treatments to that in natural controls was used as the RA index, ranging from 0 to 1. A lower RA value indicates partial RA through self‐pollination but lower yields compared with ideal outcrossing, suggesting the presence of inbreeding depression. Conversely, an RA value approaching 1 implies that seed production under complete isolation can reach levels equivalent to ideal outcrossing, demonstrating a robust RA effect.

### 2.11 Observation of Pollinating Insects and Their Behavior

Six *R. nymphaeoides* individuals with minimal environmental interference were selected, and 10 flowers per plant (totaling 60 flowers) were subjected to fixed‐point observations. From April 21 to 23, 2024 (during the peak flowering period of *R. nymphaeoides*), continuous 3‐day surveys of flower‐visiting insects were conducted daily between 08:00 and 18:00. Observations were made hourly for 30‐min intervals, during which the following parameters were recorded: insect species, landing position, whether the stigma or stamens were contacted, landing time, duration of stay, and consecutive revisits. The visitation frequency and duration of stay of major flower‐visiting insects were quantified following the methodology described by Ji et al. (2023). Visitation frequency was measured by recording the number of visits within a 5‐min window using a stopwatch, starting when an insect landed on a flower. This process was replicated 60 times. A visit was defined as any insect contact with floral structures (petals, stamens, or stigma). Duration of stay was timed from the initial landing of an insect on a flower until its departure, with 60 independent replicates. Based on the criteria established by Stout (2007), effective pollinators of *R. nymphaeoides* were identified as those insects capable of carrying pollen and able to contact the stigma. Insect‐catching nets were used to collect visiting insects. Additionally, flower‐visiting insects were collected using insect nets. To clearly document their morphological features, the captured insects were anesthetized with 75% ethanol and positioned on the central part of *R. nymphaeoides* flowers. A digital camera was used to photograph them, generating a series of images. The specimens were then transported to the laboratory, where voucher specimens were prepared and deposited in the Insect Collection of the Guizhou Academy of Forestry Sciences.

## 3 Results

### 3.1 The Flowering Phenology of *R. nymphaeoides*

**FIGURE 3:**
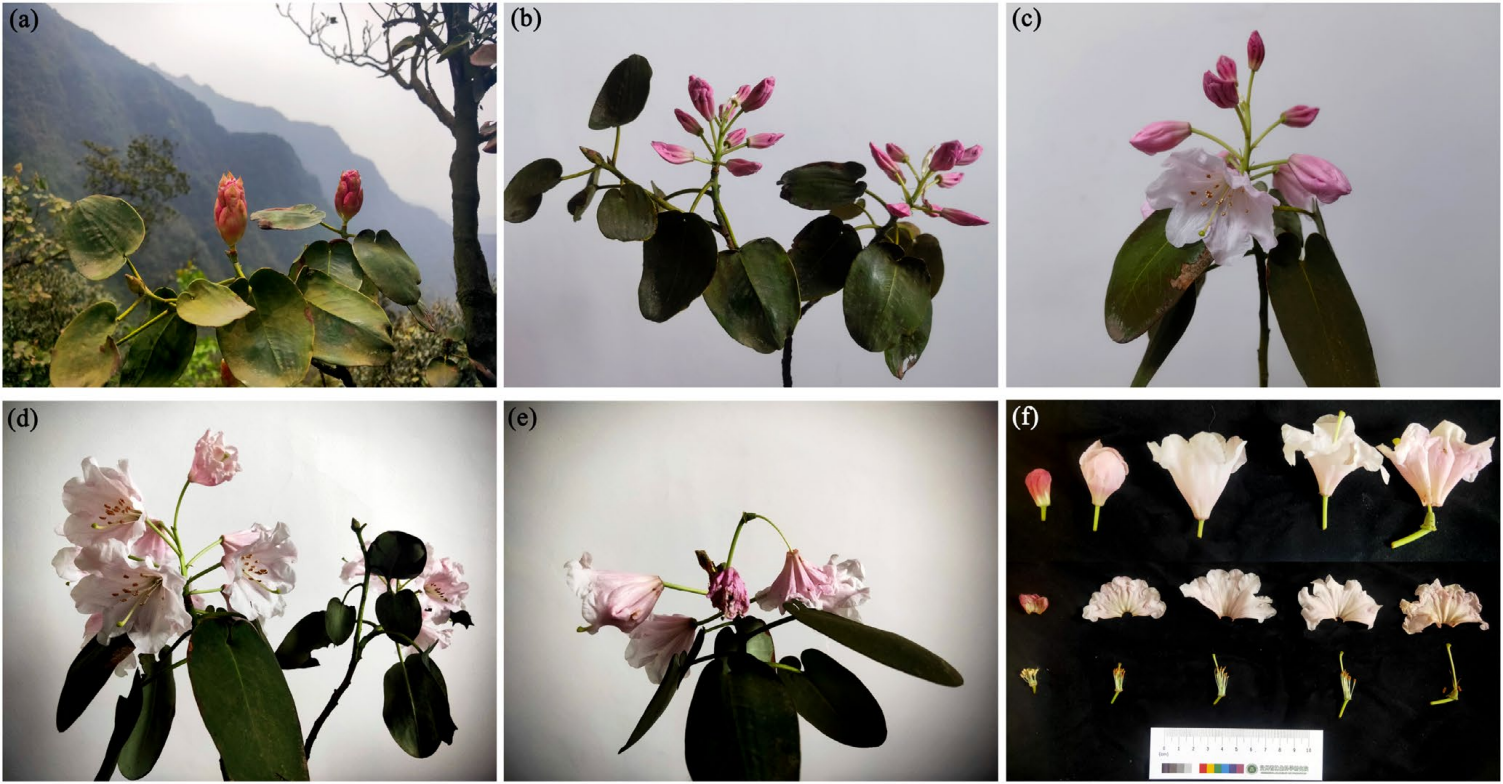
Flowering process of *R. nymphaeoides*. (a) Flower bud stage; (b) flower bud expansion stage; (c) initial flowering stage; (d) full‐bloom stage; (e) withering stage; and (f) dissection of flower parts in various stages.

**FIGURE 4:**
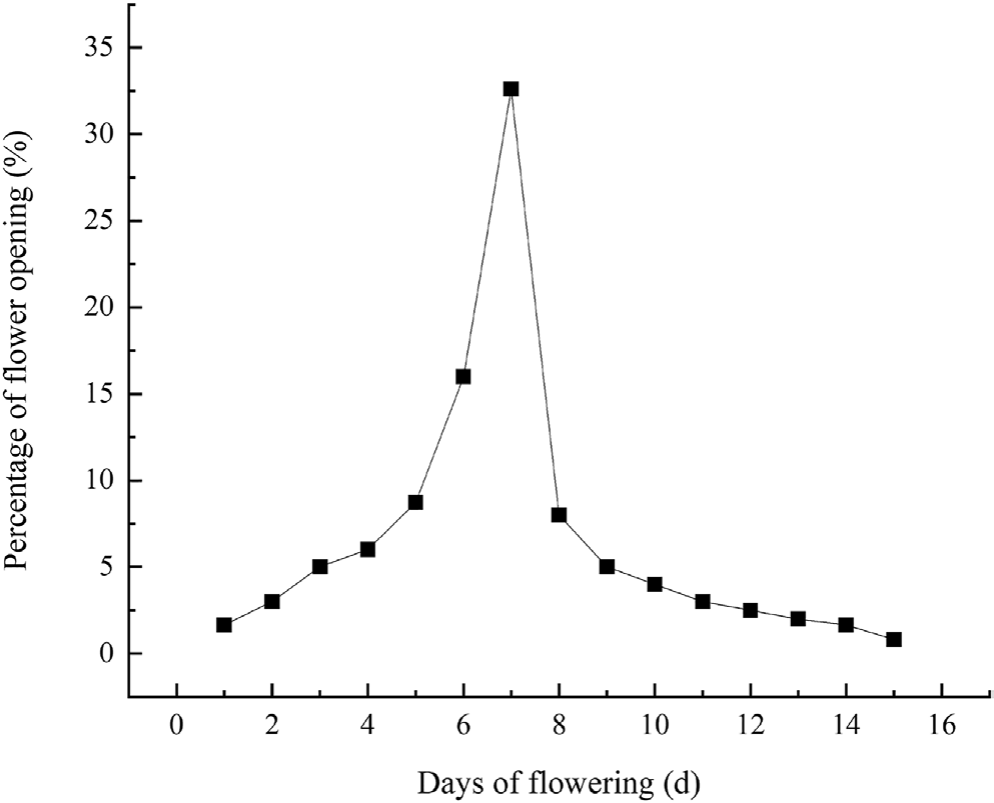
Flowering amplitude curves of *R. nymphaeoides*.

*R. nymphaeoides* has a terminal racemose inflorescence, with each inflorescence containing 7–10 flowers that are prominently displayed. As shown in Table 1, the first flowers of *R. nymphaeoides* appear in early April, with peak flowering occurring in late April and the flowering period ending in mid‐May. The duration of individual flowers is approximately 10 ± 2.69 days, and the flowering process can be divided into five stages: bud stage, bud enlargement stage, initial flowering stage, peak flowering stage, and wilting stage (Figure 3). During the bud stage, the stigma of *R. nymphaeoides* is wrapped by the petals, and the flowers are deep red. Upon dissecting the flowers, it was found that the anthers are white and immature, the filaments are relatively short, and the stigma is dark green and does not secrete any mucilage. In the bud enlargement stage, the pistil and stamens begin to separate, and the anthers turn yellow. During the initial flowering stage, the petals change to a light red color, fully open, and the anthers turn brown. In the peak flowering stage, the petals are light red, the corolla becomes white, the stigma curves into an arch shape, and mucilage is present. In the wilting stage, the edges of the petals curl and turn brown, and the stigma becomes dark brown with mucilage. The synchrony of flowering in individual plants is relatively high, with a flowering synchrony index of 0.82 ± 0.27, exhibiting a unimodal flowering process, and the number of flowers reaches its peak on the seventh day after the first flowers appear (Figure 4).

**TABLE 1:** The phenology of *R*. *nymphaeoides*: the level of the population, individual and single flower.

Observation item	Population level	Individual level	Single‐flower level
First flowering stage (day/month)	18/4	10/4	—
Duration (days)	31	26 ± 2.07	10 ± 2.69
Peak flowering date (day/month)	28/4	18/4	—
Flowering amplitude (no. of flowers)	—	3.26	—
Flowering synchrony index	—	0.82 ± 0.27	—
Last flowering date (day/month)	18/5	5/5	—

### 3.2 The Floral Characteristics of *R. nymphaeoides*

*R. nymphaeoides* has light red, wide bell‐shaped flowers with a diameter ranging from 45.08 to 86.94 mm, featuring seven lobes that are broadly rounded with notches at the tips. There are 15 stamens of varying lengths, with the shortest measuring 16.15 ± 4.55 mm and the longest measuring 24.08 ± 6.42 mm. The anthers are brown and elongated oval in shape. The main morphological indicators of the flowers of *R. nymphaeoides* are shown in Table 2. By measuring the nectar reward indicators, the average nectar volume of *R. nymphaeoides* flowers is 12.34 ± 2.21 μL, with an average sugar concentration of 21.16% ± 1.71%. The seven petals of *R. nymphaeoides* are of the same color, and the reflectance spectrum shows a peak between 320 and 400 nm, with the peak reflectance of the upper petals at 344.06 ± 1.01 nm and that of the lower petals at 354.72 ± 3.97 nm.

**TABLE 2:** Floral syndrome of *R. nymphaeoides*.

Indices	Minimum	Maximum	Mean	Standard deviation	Coefficient of variation/%
Petal length/mm	21.08	49.73	37.91	8.07	21.29
Petal width/mm	13.19	25.24	18.96	3.69	19.46
Flower diameter/mm	45.08	86.94	61.71	8.03	11.20
Length of corolla tube/mm	13.19	26.23	19.95	3.70	18.55
Stigma diameter/mm	2.26	4.60	3.31	0.67	20.24
Style length/mm	18.63	42.29	34.28	7.63	22.26
Pedicel length/mm	17.18	43.13	29.81	8.65	29.02
Length of the shortest filament/mm	7.64	22.77	16.15	4.55	28.17
Length of the longest filament/mm	11.51	32.20	24.08	6.42	26.66
Shortest distance of stigma to anthers/mm	4.65	21.85	14.23	4.96	34.86
Anther length/mm	1.86	4.64	3.15	0.75	23.81
Ovary length/mm	4.10	8.56	6.38	1.40	21.94
Ovary width/mm	3.52	6.83	5.01	0.86	17.17
Nectar volume/μL	8.25	16.38	12.34	2.21	17.91
Sugar concentration/%	17.56	24.62	21.16	1.71	8.08
The peak of the reflection spectrum of the upper petals/nm	340.85	344.90	344.06	1.01	0.29
The peak of the reflection spectrum of the lower petals/nm	351.20	364.60	354.72	3.97	1.12

### 3.3 The Pollen Morphological Characteristics of *R. nymphaeoides*

**FIGURE 5:**
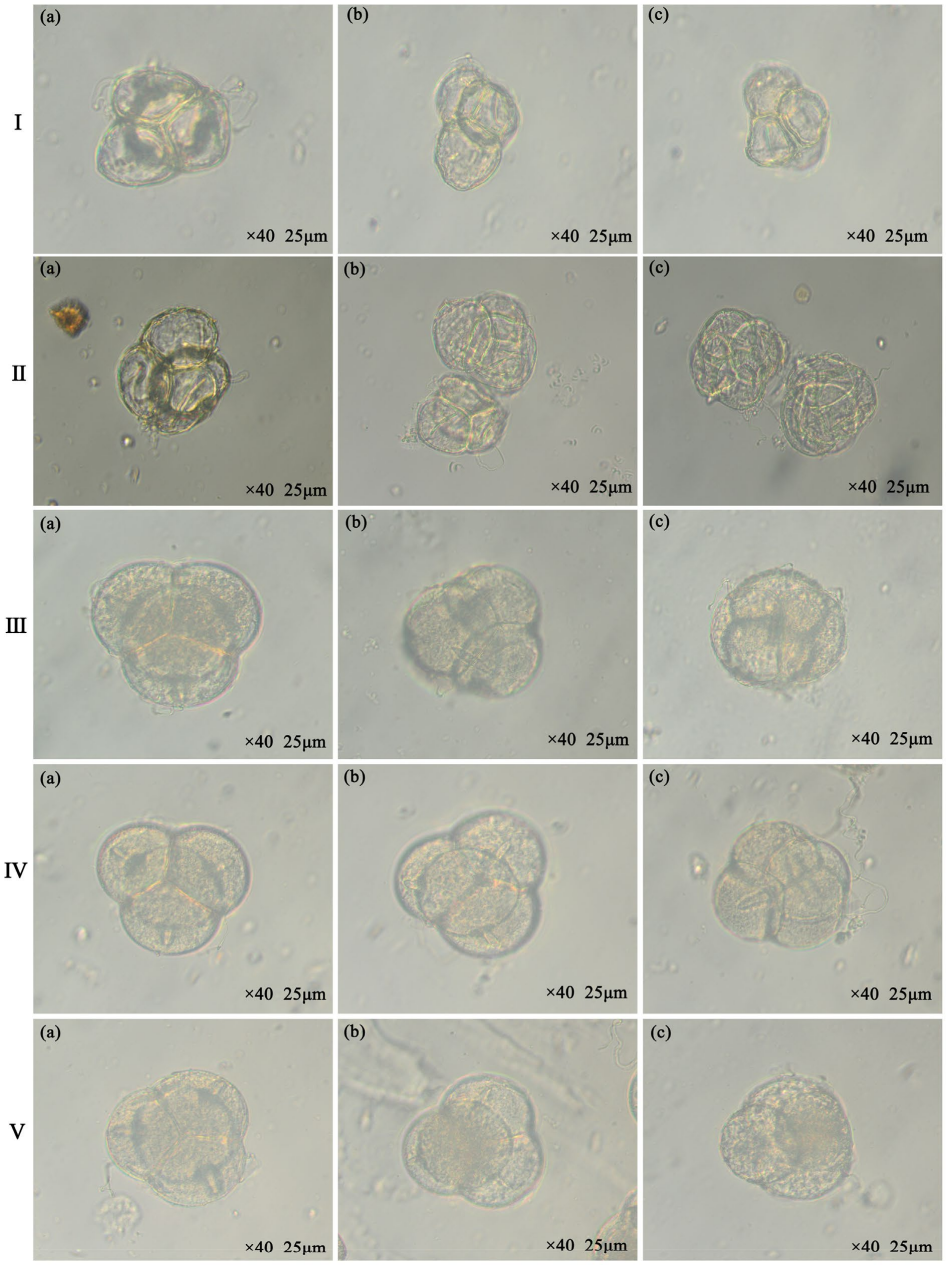
Pollen grain morphology of *R. nymphaeoides* at different development stages under an optical microscope. I (flower bud stage); II (flower bud expansion stage); III (initial flowering stage); IV (full‐bloom stage); V (withering stage); (a) polar view; (b) polar view; and (c) equatorial view.

**FIGURE 6:**
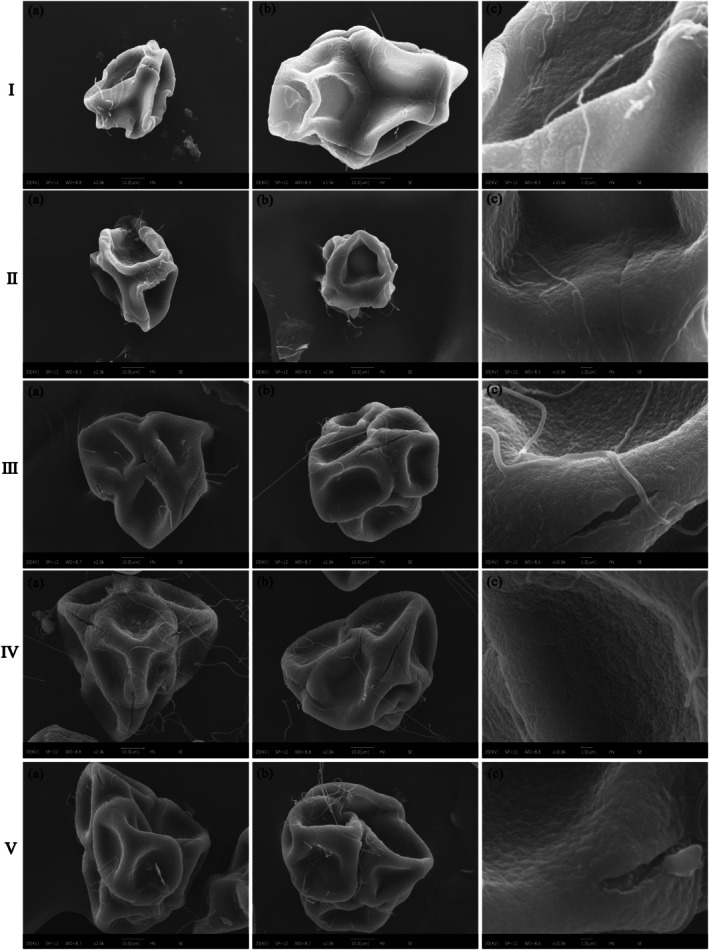
Pollen grain morphology of *R. nymphaeoides* at different development stages under scanning electron microscopy: I (Flower bud stage); II (Flower bud expansion stage); III (Initial flowering stage); IV (Full‐bloom stage); V (Withering stage); (a) polar view; (b) equatorial view; (c) exine sculpture.

Through observations using optical microscopy (Figure 5) and scanning electron microscopy (Figure 6), the pollen of *R. nymphaeoides* is identified as a compound tetrad, arranged in a regular tetrahedral formation (with one grain on top and three grains below, or three grains on top and one grain below) or in a cruciform arrangement (with two grains above and two grains below when viewed from the equatorial plane). The diameter of the tetrads ranges from 54.56 to 65.23 μm, with an average diameter of 56.47 μm. The surface of the pollen grains is covered with sticky threads, and individual pollen grains are nearly spherical, each possessing three meridional furrows. The lengths of the furrows range from 10.21 to 24.85 μm, with relative lengths between 0.24 and 0.76; the average furrow length and average relative length are 15.78 μm and 0.46, respectively. The furrow widths range from 4.86 to 9.74 μm, with an average of 6.85 μm. The furrows on adjacent pollen grains are interconnected and communicate with each other, typically being flat at the end closest to the adjacent pollen grain and tapering towards the apex of the pollen grain (Figure 5I‐c). The edges of the furrows are thickened, with internal contents exposed, and the inter‐grain areas are uniformly granular with a rough outer wall. The granules surrounding the furrows are densely arranged in a wavy protrusion, distinctly different from the outer wall's texture (Figure 5IV‐c).

There are certain differences in the structural characteristics of pollen at different developmental stages of the flowers. In the bud stage, the tetrad pollen begins to take shape, with the central part of the pollen grains being depressed and containing very few internal contents, exhibiting high transparency under optical microscopy (Figure 5I‐a,b,c). In the bud enlargement stage, the three germination furrows are visible as rudimentary forms; the inter‐tetrad seams are shallow, and the inter‐grain areas are rough and granular, with clear cracks; the textures around the furrows are not prominent (Figure 6II‐a,b,c). In the initial flowering stage, the pollen shape becomes clearer, appearing as acute triangles when arranged in a regular tetrahedral formation, with individual pollen grains being oval‐shaped. The inter‐tetrad seams are shallower, and loose internal contents begin to appear within the germination furrows; the inter‐grain areas are uniformly coarse‐granular, and the edges of the furrows start to thicken (Figure 6III‐a,b,c). During the peak flowering stage, the pollen matures, becoming plump and appearing as rounded triangles when arranged in a regular tetrahedral formation; individual pollen grains are nearly spherical, the inter‐tetrad seams are deep, and the germination furrows are filled with internal contents. The inter‐grain areas are uniformly fine‐granular, and the edges of the furrows are thickened and exhibit wavy protrusions (Figure 6IV‐a,b,c). In the wilting stage, the pollen is filled with internal contents, appearing relatively turbid under optical microscopy (Figure 5V‐a,b,c).

Under optical microscopy, the outer wall of *R. nymphaeoides* pollen is observed to consist of two layers, with a thickness of approximately 1.5–2.0 μm (Figure 5V‐a,b,c). Under scanning electron microscopy, a blurred fine net‐like sculpture is visible on the surface of the pollen outer wall (Figure 6IV‐c).

### 3.4 Pollen Viability and Stigma Receptivity of *R. nymphaeoides*

**FIGURE 7:**
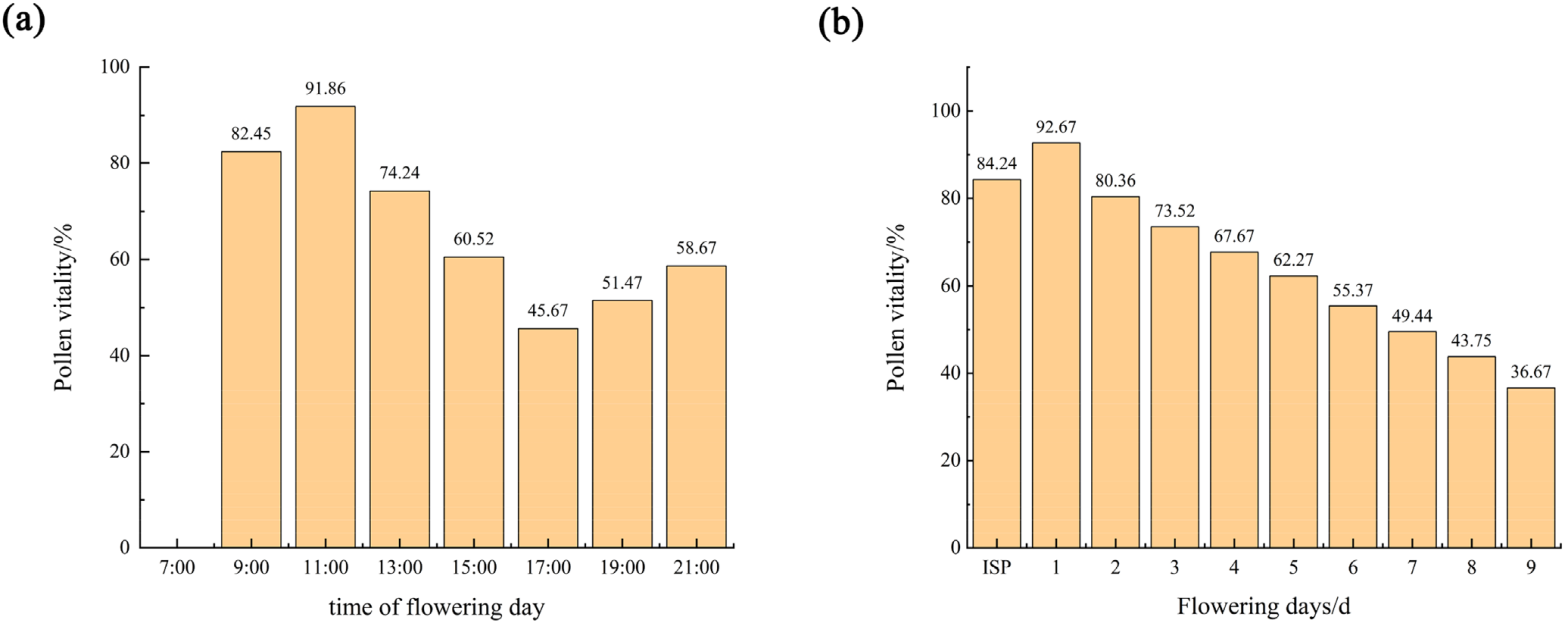
Change of pollen viability at anthesis of *R. nymphaeoides*. (a) Change of pollen viability on the first day of anthesis; (b) pollen viability on different days of anthesis, ISP: Flower bud stage (mainly refers to 1~2 days before flowering).

As shown in Figure 7, on the day of flowering, the pollen viability of *R. nymphaeoides* was highest at 11:00 a.m. (91.86%), after which it gradually decreased, reaching its lowest point at 5:00 p.m. (45.67%), and then slowly increased again. The pollen viability during the bud stage was 84.24%. As the flowers opened and the anthers dehisced, the pollen viability increased, peaking on the first day of flowering at 92.67%. After that, the pollen viability declined daily, with the color of the anthers and stigma gradually turning black. As the pollen was dispersed, its viability gradually decreased, remaining at about 36.67% by the wilting stage (the ninth day of flowering). This indicates that under natural conditions, *R. nymphaeoides* can maintain relatively high pollen viability for an extended period, which is beneficial for subsequent hybrid breeding efforts.

**FIGURE 8:**
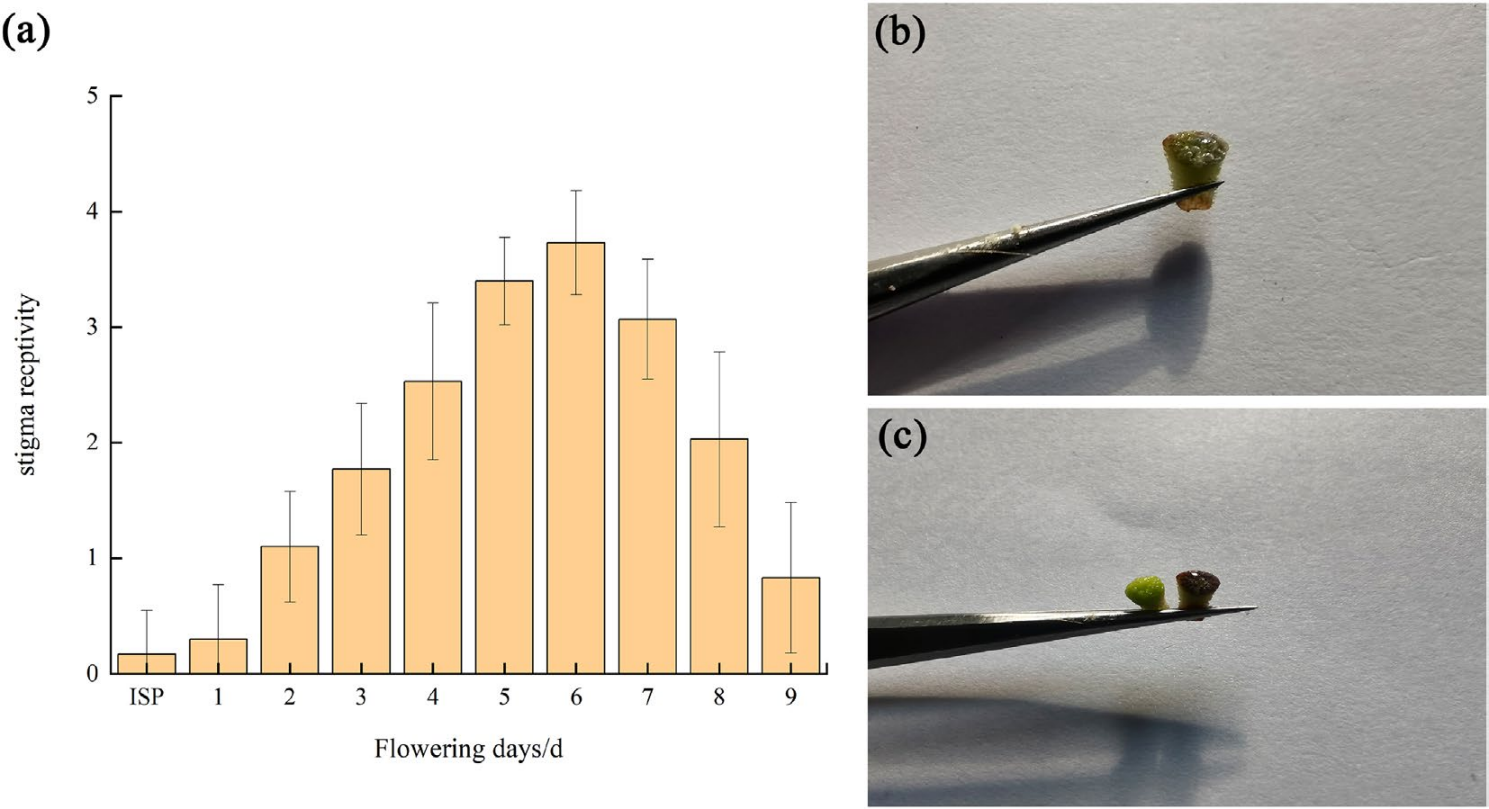
Change of stigma receptivity at anthesis of *R. nymphaeoides*. (a) Stigma receptivity in different days of anthesis, ISP: Flower bud stage (mainly refers to 1~2 days before flowering); (b) the situation when stigma produces bubbles; and (c) the situation when stigma produces bubbles and turns blue.

As shown in Figure 8, during the bud stage and on the first day of flowering, the stigmas of *R. nymphaeoides* secreted minimal mucus, exhibited faint blue coloration reactions, and demonstrated extremely weak receptivity; from days 2 to 5 post‐anthesis, the reaction between the stigmas and the test solution gradually intensified, with increasingly distinct blue coloration and a rising number of bubbles; on day 6, the reaction reached its peak intensity, characterized by the highest bubble production and strongest receptivity; on days 7 and 8, the reaction progressively weakened, accompanied by fading blue coloration and fewer bubbles; by day 9 of flowering, field observations revealed that petals and stamens of *R. nymphaeoides* had begun to abscise, and receptivity tests showed diminished color reactions and sparse bubble formation, indicating a sharp decline in stigma receptivity at this stage.

### 3.5 The Effects of Three Factors: Sucrose, H_3_BO_3_ , and CaCl_2_ on the Pollen Germination of *R. nymphaeoides*

The experimental observations revealed that most pollen on the culture media began to germinate after 4 h. After 9 h of cultivation, the maximum pollen germination was reached on media 2 and 6, while on media 22 and 25, the number of germinated pollen grains only stabilized after 26 h (Table 3). The pollen germination rates of the various combinations in the L_25_(5^3^) orthogonal experiment showed significant differences (*p* < 0.05), with the highest germination rate (92.62%) achieved using the medium formulation of 150 g·L^−1^ sucrose + 200 mg·L^−1^ H_3_BO_3_ + 50 mg·L^−1^ CaCl_2_ after 17 h of cultivation. Range analysis of the data in Table 3 indicated that the order of importance of the three factors was as follows: sucrose → CaCl_2_ → H_3_BO_3_, with ranges of 47.10, 21.26, and 15.56, respectively. The optimal factor for sucrose was A3 (100 g·L^−1^), for H_3_BO_3_ it was B4 (200 mg·L^−1^), and for CaCl_2_ it was C2 (50 mg·L^−1^). This indicates that the suitable combination of sucrose and H_3_BO_3_ concentrations is most beneficial for the pollen germination of *R. nymphaeoides*. A small amount of CaCl_2_ can promote pollen germination, but as the concentration increases, it significantly inhibits germination. Sucrose is identified as the determining factor affecting the pollen germination rate of *R. nymphaeoides*, showing a trend of increasing germination rate followed by a decrease as its concentration rises, with 100 g·L^−1^ being the optimal concentration. When the concentration reaches 300 g·L^−1^, the pollen germination rate declines, the time required for germination is extended, and some media exhibit ruptures at the tips of the pollen tubes. Although the promoting effect of H_3_BO_3_ on germination is not as significant as that of sucrose, it does contribute to pollen germination to some extent. Furthermore, sucrose, H_3_BO_3_, and CaCl_2_ are not essential conditions for the pollen germination of *R. nymphaeoides*, as a germination rate of 23.68% was still observed in a medium containing only 10 g·L^−1^ agar.

**TABLE 3:** The germination rate of *R*. *nymphaeoides* pollen grains in the L_25_ (5^3^) orthogonal experiment.

No.	Sucrose/(g·L^−1^)	H_3_BO_3_/(mg·L^−1^)	CaCl_2_/(mg·L^−1^)	Germination rate/%
1	0	0	0	23.68 ± 1.67^c^
2	0	50	50	70.24 ± 1.34^mn^
3	0	100	150	55.34 ± 1.22^j^
4	0	200	250	73.28 ± 1.42^n^
5	0	300	350	40.26 ± 1.63^g^
6	50	0	50	76.67 ± 1.44^o^
7	50	50	150	68.35 ± 2.16^lm^
8	50	100	250	61.82 ± 1.33^k^
9	50	200	350	67.25 ± 1.56^lm^
10	50	300	0	72.21 ± 1.52^n^
11	100	0	150	76.38 ± 1.03^o^
12	100	50	250	84.78 ± 1.45^p^
13	100	100	350	43.52 ± 1.48^h^
14	100	200	0	79.43 ± 1.64^q^
15	100	300	50	86.85 ± 0.92^lm^
16	150	0	250	51.05 ± 0.82^i^
17	150	50	350	65.58 ± 1.02^l^
18	150	100	0	86.14 ± 1.57^pq^
19	150	200	50	92.62 ± 2.05^pq^
20	150	300	150	36.94 ± 1.74^e^
21	300	0	350	41.72 ± 1.58^g^
22	300	50	0	19.22 ± 1.47^b^
23	300	100	50	38.54 ± 1.41^f^
24	300	200	150	14.20 ± 0.56^a^
25	300	300	250	27.57 ± 1.63^d^

*Note:* Different small letters in each column indicate significant difference at *p* < 0.05 level.

### 3.6 The Effects of Different Storage Temperatures on the Pollen Viability of *R. nymphaeoides*

**FIGURE 9:**
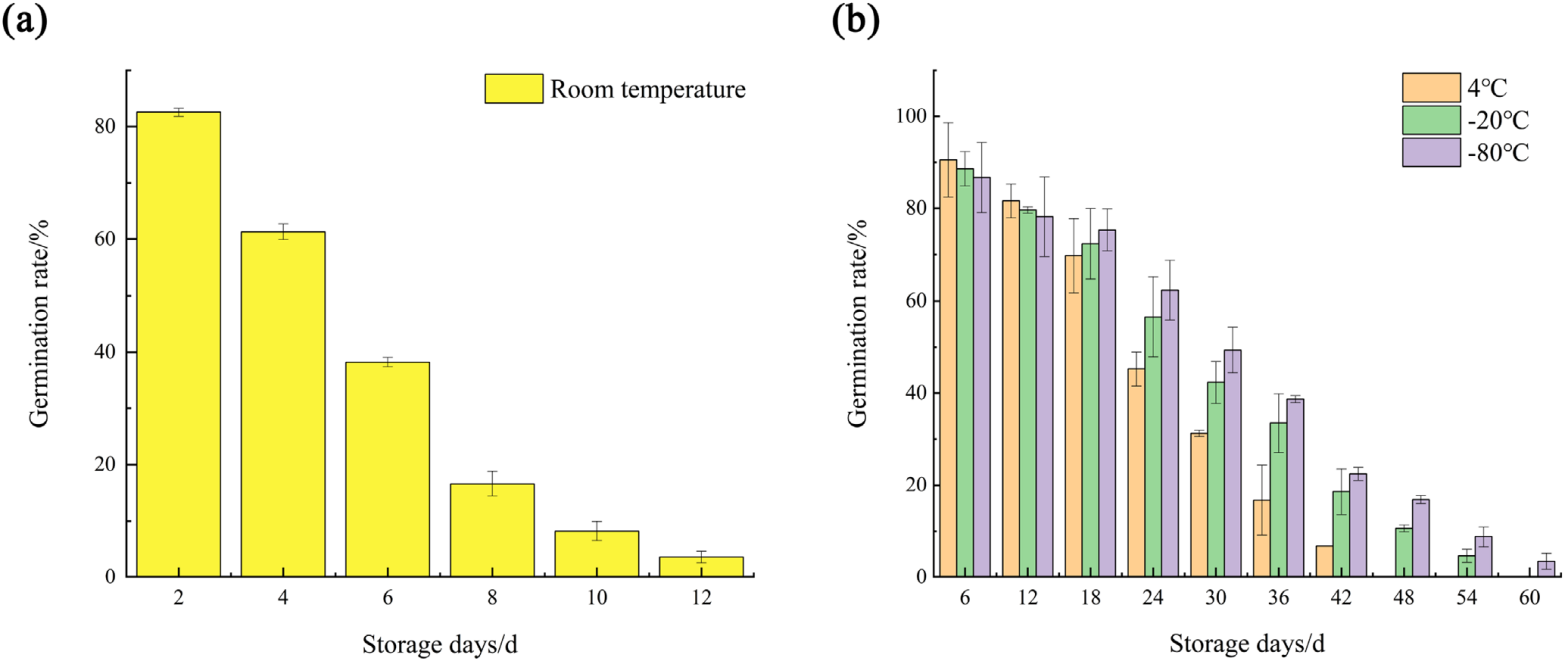
(a) Change of germination rate of *R. nymphaeoides* pollen grains at room temperature storage; (b) change of germination rate of *R. nymphaeoides* pollen grains at 4°C, −20°C and −80°C temperature storage.

The experiment measured the pollen germination rates of *R. nymphaeoides* at four storage temperatures: room temperature (20°C–25°C), 4°C, −20°C, and −80°C, over different storage durations. The results indicated (Figure 9) that with the increase in storage days, the pollen germination rates at all four temperatures showed a gradual declining trend. Under room temperature conditions, the pollen germination rate of *R. nymphaeoides* decreased most rapidly, dropping to a low level (16.62%) by the eighth day, and by the 12th day, the germination rate fell below 5%, making it essentially unusable for hybridization as a pollen parent. At 4°C, −20°C, and −80°C, the decline in germination rates was slower. During the first 12 days of storage, the 4°C condition showed slightly better preservation effects, but by the 24th day, there was a significant drop in germination rate, which remained at 16.72% by the 36th day. Pollen stored at −20°C and −80°C did not show a significant decline until the 42nd day, with the pollen stored at −80°C still having a germination rate of 8.76% by the 54th day. Therefore, the optimal storage temperature for *R. nymphaeoides* pollen is −80°C, followed by −20°C, indicating that certain low temperatures are beneficial for extending pollen longevity.

### 3.7 Pollen Ovule Ratio and Hybridization Index

According to the criteria established by Dafni and Maués (1998), the hybridization index of *R. nymphaeoides* was estimated. The corolla diameter of *R. nymphaeoides* is 61.71 ± 8.03 mm, which is greater than 6 mm, thus recorded as 3. It possesses both pistils and stamens, with the stamens maturing first, recorded as 1. During flowering, the stigma is curved and positioned above the anthers, creating spatial separation, which is also recorded as 1. Therefore, the hybridization index of *R. nymphaeoides* is calculated as 3 + 1 + 1 = 5, indicating that the breeding system of *R. nymphaeoides* is obligate cross‐fertilization.

The number of pollen grains per flower of *R. nymphaeoides* is 497,689 ± 3423.57, and the number of ovules per flower is 726.48 ± 52.76. Thus, the pollen–ovule ratio is 685.07 ± 54.88. Based on the evaluation criteria for breeding systems by Cruden (1977), it can be determined that the breeding system of *R. nymphaeoides* is facultative cross‐fertilization.

### 3.8 The Effects of Different Pollination Methods on the Fruit Set Rate of *R. nymphaeoides*

As shown in Table 4, the natural pollination group produced 13 fruits with a fruit set rate of 43.33%, indicating that *R. nymphaeoides* can achieve natural fruit set in wild populations in Gulin County, Sichuan Province, although the efficiency is relatively low. Direct bagging resulted in an average fruit set rate of 3.33%, demonstrating the absence of autonomous self‐pollination. This aligns with its morphological trait of a large stigma‐anther separation distance (14.23 ± 4.96 mm). Emasculated and bagged treatments yielded no fruits (0%), confirming that *R. nymphaeoides* does not exhibit parthenocarpy. Emasculation not bagged showed slightly lower fruit numbers and fruit set rates compared with natural controls, suggesting that *R. nymphaeoides* relies on pollinators for cross‐pollination, yet achieves near‐natural fruit set levels under insect‐mediated conditions. Artificial self‐pollination produced 23 fruits (76.67%), demonstrating partial self‐compatibility in *R. nymphaeoides*. Different strains' cross‐pollination achieved a higher fruit set rate (83.33%) than strains' cross‐pollination (73.33%), both significantly exceeding natural controls. This further supports the effectiveness of outcrossing mechanisms in *R. nymphaeoides*, with a predominant reliance on cross‐pollination, while fruit production is limited by pollinator availability. These results collectively suggest that *R. nymphaeoides* exhibits a facultative xenogamous mixed mating system dependent on pollinators.

**TABLE 4:** Fruit set of *R*. *nymphaeoides* under different pollination treatments.

Pollination treatments	Number of inflorescences	Number of flowers	Number of fruits	Fruit set/%
Natural pollination	10	30	13	43.33
Direct bagging	10	30	1	3.33
Emasculated and bagged	10	30	0	0.00
Emasculation is not bagged	10	30	11	36.67
Artificial self‐pollination	10	30	23	76.67
Strains cross‐pollination	10	30	22	73.33
Different strains cross‐pollination	10	30	25	83.33

A generalized linear model (GLM) with a binomial distribution and logistic link function was applied to analyze the fruit set rates between natural pollination and different strains cross‐pollination treatments in *R. nymphaeoides*. The results showed significant differences (Wald‐*χ* ^2^ = 964.67, df = 3, *p* < 0.001), indicating that there are limitations to pollination, with a pollination limitation index (PL) of 0.33. The RA coefficient for *R. nymphaeoides* is 0.15, indicating that it relies on cross‐pollination and has weak RA capabilities.

The above results indicate that the breeding system of *R. nymphaeoides* is a facultative cross‐fertilization mixed mating system that requires pollinators.

### 3.9 Pollinating Insects and Their Behavior

**FIGURE 10:**
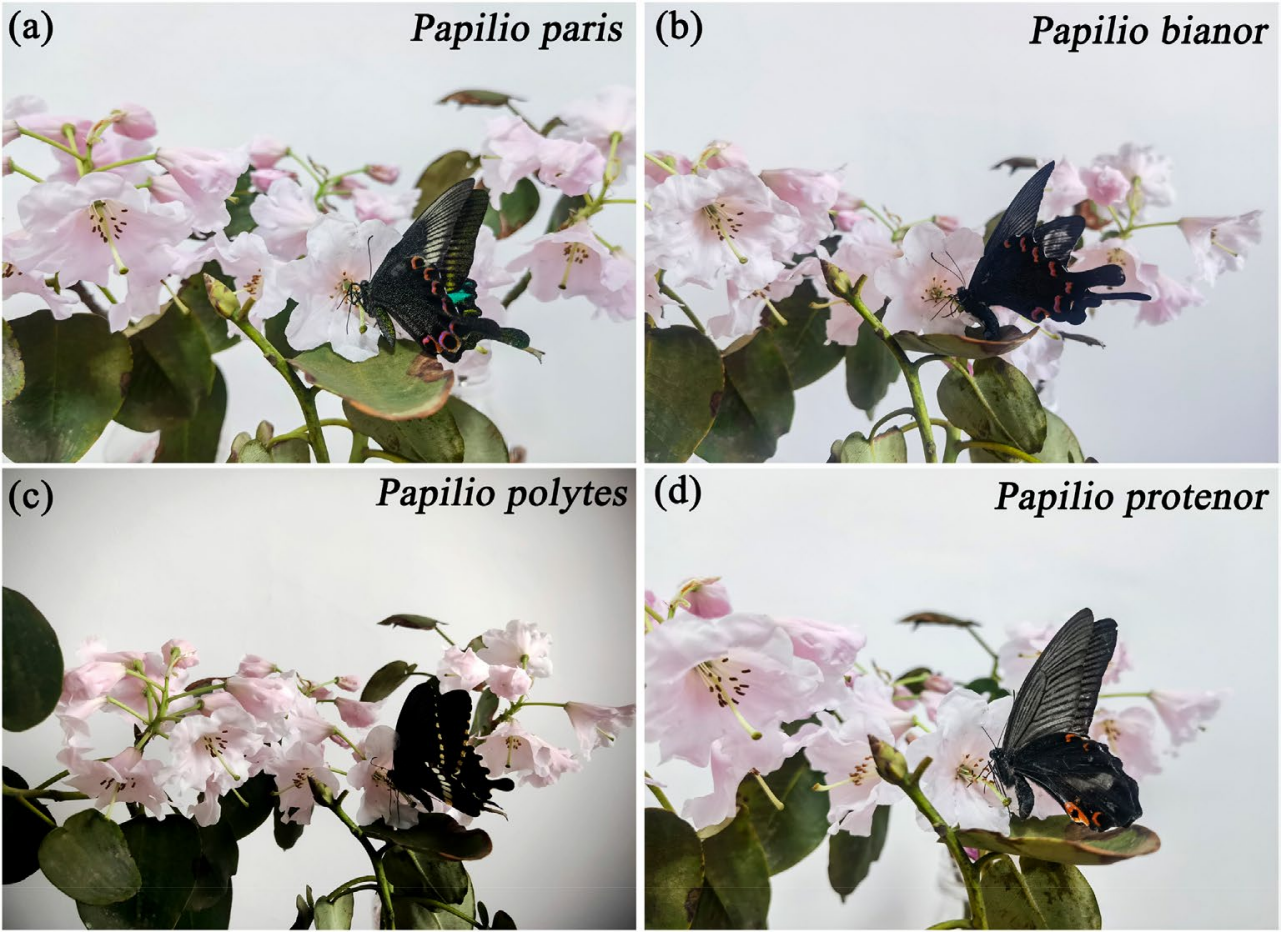
The main visiting insects are Lepidoptera of *R. nymphaeoides* (after capturing the dizziness, place it on a flower for shooting). (a) *Papilio paris*; (b) *Papilio bianor*; (c) *Papilio polytes*; (d) *Papilio protenor.*

**FIGURE 11:**
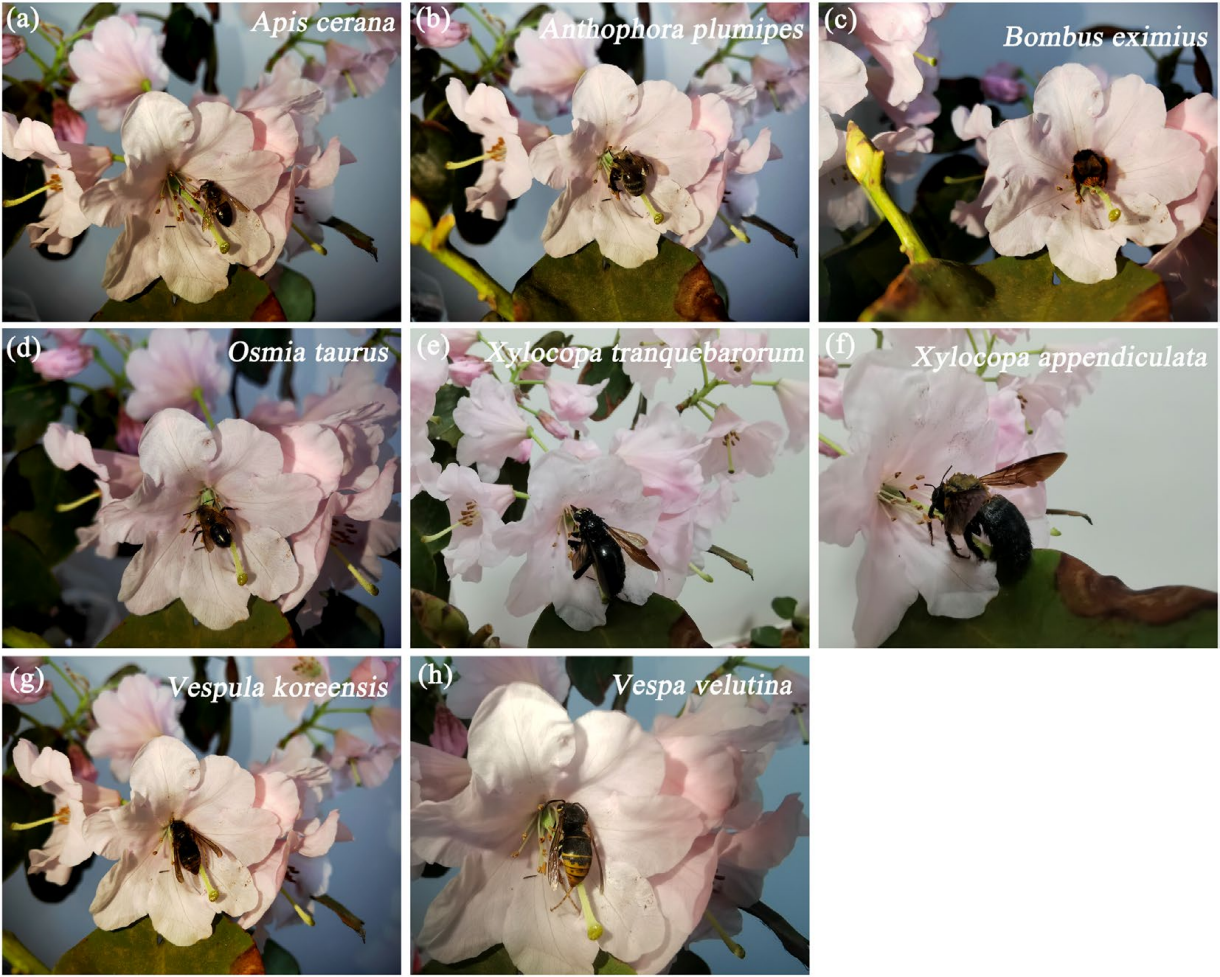
The main visiting insects are Hymenoptera of *R. nymphaeoides* (after capturing the dizziness, place it on a flower for shooting). (a) *Apis cerana*; (b) *Anthophora plumipes*; (c) *Bombus eximius*; (d) *Osmia taurus*; (e) *Xylocopa tranquebarorum*; (f) *Xylocopa appendiculata*; (g) *Vespula koreensis*; (h) *Vespa velutina*.

**FIGURE 12:**
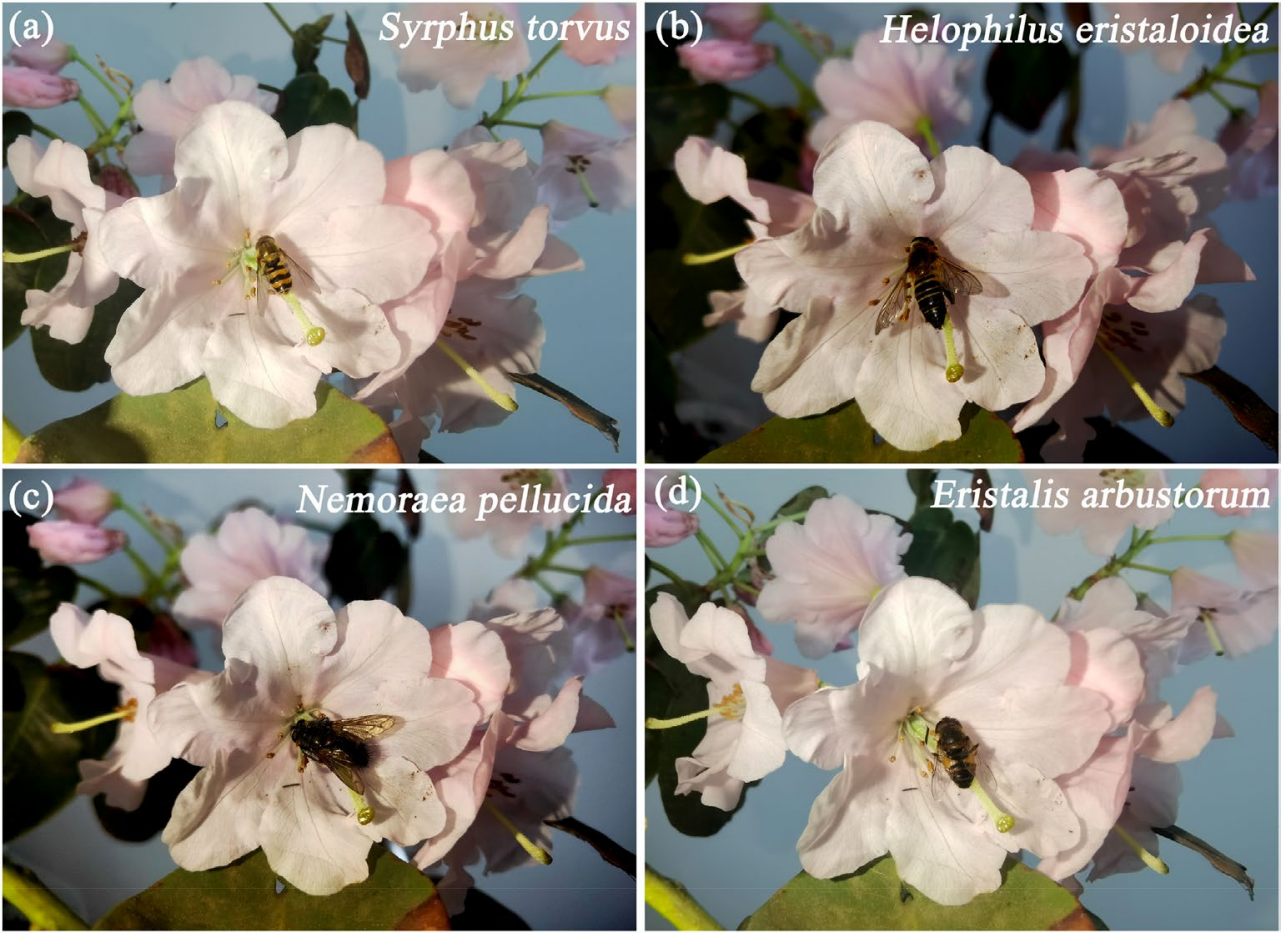
The main visiting insects are Diptera of *R. nymphaeoides* (after capturing the dizziness, place it on a flower for shooting). (a) *Syrphus torvus*; (b) *Helophilus eristaloidea*; (c) *Nemoraea pellucida*; (d) *Eristalis arbustorum*.

Through observations, a total of 16 species of pollinating insects for *R. nymphaeoides* were identified, including *Papilio paris*, *Papilio bianor*, *Papilio polytes*, and *Papilio protenor* from the family Papilionidae (Figure 10); *Syrphus torvus* , *Helophilus eristaloidea*, and *Eristalis arbustorum* from the family Syrphidae; *Nemoraea pellucida* from the family Tachinidae (Figure 11); *Apis cerana* , *Bombus eximius* , *Xylocopa appendiculata* , *Xylocopa tranquebarorum* , and *Anthophora plumipes* from the family Apidae; *Osmia taurus* from the family Megachilidae; and *Vespula koreensis* and *Vespa velutina* from the family Vespidae (Figure 12). Among these, *P. paris* , *P. bianor*, *P. polytes*, and *P. protenor* are effective pollinators, as their wings and abdomens were clearly observed to carry pollen. When they landed on the flowers of *R. nymphaeoides*, their wings touched the stigma, or their abdomens were pressed against the stigma, while they extended their proboscis into the floral tube. The proboscis length of the tested *P. paris* was 19.26 mm, which is approximately equal to the length of the corolla tube of *R. nymphaeoides*. *A. cerana* , *V. koreensis* , *V. velutina* , *X. appendiculata* , and *X. tranquebarorum* are also effective pollinators. *A. cerana* , *V. koreensis* , and *V. velutina* hovered in the air before visiting flowers, then landed on a specific plant to forage. When they landed on the corolla of *R. nymphaeoides*, their heads extended into the floral cavity to feed on nectar or pollen, rotating their bodies and shifting their hind legs, moving relatively slowly and staying on the same flower for several minutes. During this process, they collected a large amount of pollen, effectively pollinating *R. nymphaeoides*. When *X. appendiculata* and *X. tranquebarorum* approached the flowers of *R. nymphaeoides*, they vibrated their wings while hovering, first touching the corolla with their forelegs, then collecting pollen, and shaking to ensure that pollen adhered to their heads and bodies. They used their legs to brush and gather the pollen into their pollen baskets; when they moved to another flower, they transferred the pollen they carried to the stigma, effectively pollinating *R. nymphaeoides*. Small insects such as *B. eximius* , *A. plumipes* , *S. torvus* , *H. eristaloidea*, *E. arbustorum* , and *N. pellucida* acted as nectar robbers, as they did not carry pollen on their bodies and did not contact the stigma. *O. taurus* were identified as pollen thieves, spending a long time visiting flowers and only flying to the next flower after collecting all the pollen from the 15 stamens of one flower. Additionally, no other visiting animals were observed.

Through three consecutive days of observation (Table 5), *O. taurus* exhibited the highest visitation frequency, averaging 6.91 visits·min^−1^, followed by *B. eximius* with 5.76 visits·min^−1^. Insects such as *A. cerana* , *V. koreensis* , *V. velutina* , *X. appendiculata* , and *X. tranquebarorum* showed moderate visitation frequencies clustered between 3.65 and 4.32 visits·min^−1^, while smaller insects including *A. plumipes* , *S. torvus* , *H. eristaloidea*, *E. arbustorum* , and *N. pellucida* displayed intermediate frequencies ranging from 2.11 to 2.57 visits·min^−1^. The lowest visitation frequencies were observed in *P. paris* , *P. bianor*, *P. polytes*, and *P. protenor*, concentrated at 1.34~2.08 visits·min^−1^. Regarding flower residence time, *O. taurus* spent the longest average duration on flowers (11.30 s), with individual visits reaching up to 25 s, followed by *B. eximius* at 9.21 s. *A. cerana* , *V. koreensis* , *V. velutina* , *X. appendiculata* , and *X. tranquebarorum* averaged 7.21~7.84 s, whereas smaller insects like *A. plumipes* , *S. torvus* , *H. eristaloidea*, *E. arbustorum* , and *N. pellucida* showed intermediate durations of 5.33~5.92 s. The shortest residence times were recorded for *P. paris* , *P. bianor*, *P. polytes*, and *P. protenor*, ranging from 3.42 to 5.24 s.

**TABLE 5:** Visitation frequency and duration of stay of major flower‐visiting insects in *R. nymphaeoides*.

Flower‐visiting insects	The number of individual insects	The number of flowers visited	Visitation frequency (times·min^−1^)	Average visitation frequency (times·min^−1^)	Duration of stay(s)	Average duration of stay(s)
*Papilio paris*	3	26	1~3	2.08 ± 0.38	3~18	4.63 ± 0.02
*Papilio bianor*	2	18	1~2	1.62 ± 0.25	3~6	3.42 ± 0.14
*Papilio polytes*	1	15	1~2	1.34 ± 0.26	3~8	3.89 ± 0.76
*Papilio protenor*	2	12	1~3	2.03 ± 0.53	4~15	5.24 ± 1.02
*Syrphus torvus*	1	14	1~4	2.18 ± 0.99	4~8	5.67 ± 1.12
*Helophilus eristaloidea*	1	10	1~4	2.22 ± 1.03	5~8	5.83 ± 1.34
*Eristalis arbustorum*	3	24	1~5	2.57 ± 1.11	4~9	5.33 ± 0.57
*Nemoraea pellucida*	1	9	1~4	2.11 ± 1.42	5~12	5.92 ± 0.42
*Apis cerana*	5	48	2~5	4.32 ± 0.47	7~16	7.84 ± 1.23
*Bombus eximius*	3	36	3~7	5.76 ± 1.22	8~21	9.21 ± 1.76
*Xylocopa appendiculata*	2	19	2~5	3.84 ± 0.56	7~14	7.67 ± 0.93
*Xylocopa tranquebarorum*	1	8	2~4	3.65 ± 0.32	6~10	7.33 ± 2.46
*Anthophora plumipes*	1	4	1~4	2.37 ± 1.07	4~7	5.42 ± 0.33
*Osmia taurus*	2	28	4~10	6.91 ± 1.80	8~25	11.30 ± 1.67
*Vespula koreensis*	1	4	2~5	3.76 ± 0.77	6~15	7.21 ± 1.28
*Vespa velutina*	1	6	2~5	3.97 ± 0.65	6~12	7.58 ± 0.22

Additionally, it was observed that the pollination activities of the pollinators may be significantly influenced by natural factors. During the flowering period of *R. nymphaeoides*, the frequency of visitors was higher when temperatures were warmer and the weather was clear, primarily due to the prominent display and fragrant scent of the flowers, which attracted pollinators. Conversely, during the peak flowering period, frequent rainy days, low humidity, and lower temperatures negatively affected the activity frequency of the visitors.

## 4 Discussion

### 4.1 Flowering Phenology and Floral Characteristics of *R. nymphaeoides*

Flowers are the reproductive organs of angiosperms, and their main function is to facilitate sexual reproduction (Liu et al. 2022). Flowering characteristics are one of the important factors affecting plant fitness, primarily including the number of flowers, flowering time, and duration of flowering (Wu et al. 2021). These characteristics not only influence the reproductive success of individuals and populations (Weiss 1991) but also affect the quantity, types, and foraging behaviors of pollinators (Dai and Tan 2011). In this study, the first flowers of *R. nymphaeoides* appeared in early April, with peak flowering occurring in late April and the flowering period ending in mid‐May. During this period, temperatures were consistently above 20°C, and relative humidity was below 60%, providing favorable environmental conditions for flowering and fruiting of this species. The flowering process of individual flowers can be divided into five stages: bud stage, bud enlargement stage, initial flowering stage, peak flowering stage, and wilting stage, exhibiting a phenomenon of synchronous flowering. According to Herrera (1986), this flowering pattern is referred to as a “mass‐flowering pattern,” which is beneficial for rapidly increasing flower display within a relatively concentrated flowering period, attracting more pollinators and increasing opportunities for cross‐pollination (Zhang and Qiu 2017; Ye et al. 2022). Therefore, the flowering characteristics of *R. nymphaeoides* are more conducive to its adaptation to adverse environments. This result is consistent with findings from studies on other *Rhododendron* species, such as *R. hemsleyanum* (Xie et al. 2023), *R. griersonianum* (Liu et al. 2025), and *R. longipedicellatum* (Li et al. 2018).

Floral syndromes are adaptive strategies that plants exhibit in response to pollinators as a result of long‐term evolutionary processes (Goodwillie et al. 2010). In this study, *R. nymphaeoides* exhibited a large corolla with pale‐red flowers arranged in umbel inflorescences, characterized by an intense fragrance, unfolded petals, and full floral display during blooming. The average nectar volume of *R. nymphaeoides* was 12.34 ± 2.21 μL, with a sugar concentration of 21.16% ± 1.71%, which is significantly higher than that of *Gentiana crassicaulis* (1.38 μL, 17.24%) (Zhang et al. 2025), comparable to its congener *R. griersonianum* (12.57 μL, 20.95%) (Liu et al. 2025), and far lower than *Punica granatum* (64.09 μL, 48.44%) (Yu et al. 2013). Studies indicate that bees‐pollinated flowers typically have sugar concentrations of 30%~50%, while birds‐pollinated flowers exhibit lower concentrations (20%) (Niu et al. 2025). Based on these thresholds, the nectar reward of *R. nymphaeoides* aligns with its reproductive strategy, catering to the pollination needs of small‐ to medium‐sized insects. The flowering pattern influences the method of pollen dispersal by attracting pollinators, thereby affecting the plant's mating system (Tang and Han 2007). This is consistent with the view of Zhang et al. (2019) who suggest that the unique floral characteristics of flowering plants serve as important visual signals to attract pollinators. Additionally, the lifespan of individual flowers of *R. nymphaeoides* is approximately 10 ± 2.69 days, with pollen being released after flowering. The duration of pollen viability and stigma receptivity is relatively long, with pollen viability remaining above 40% at peak receptivity, which is beneficial for pollen output and stigma acceptance, ensuring successful pollination. Field observations revealed that there is a brief period during which both stamens and pistils are expressed simultaneously; however, during this period, the style of *R. nymphaeoides* is curved and suspended, leading to spatial separation between the stamens and pistils. Based on the results of self‐pollination, it can be inferred that this spatial separation effectively avoids the possibility of self‐pollination. The floral characteristics of hermaphroditic plants that ensure successful cross‐pollination include the spatial separation of male and female structures, which can prevent self‐fertilization and interference with sexual function (Bhardwaj and Eckert 2001; Anderson et al. 2003). Additionally, the suspended shape of the style in *R. nymphaeoides* effectively prevents rainwater from washing away the pollen and surface mucilage on the stigma, which is consistent with previous research findings (Liu et al. 2004; Sun et al. 2008).

### 4.2 Pollen Morphology and Characteristics of *R. nymphaeoides*

The palynological research on the genus *Rhododendron* began in the mid‐20th century and has rapidly developed with the application of optical microscopy and scanning electron microscopy. Mao et al. (2000) observed the pollen of six species, one variety, and one form of *Rhododendron* from Northeast China, indicating that their morphology exhibits interspecific differences, which can be used for taxonomic studies. Gao et al. (2002a, 2002b) analyzed the pollen of 25 species from the subgenus *Azaleastrum* and four subgenera of *Rhododendron*, as well as one species from the genus *Tsuga*, concluding that pollen morphology cannot serve as a primary characteristic for distinguishing subgenera within *Rhododendron*, but it holds significant taxonomic importance for the classification of groups or subgroups. Minor differences in pollen grain size, surface ornamentation, and the length of furrows can provide useful palynological evidence for the taxonomic treatment of certain species. The observations of the pollen morphology of *R. nymphaeoides* in this study indicate that from the bud enlargement stage to the full blooming stage, the pollen gradually matures and forms tetrads, with a surface covered in sticky threads that cause the pollen grains to adhere together in the anthers. The diameter of the tetrads ranges from 26.38 to 66.40 μm, as reported by Gao et al. (2002a) and the length‐to‐width ratio of the germination furrows is slightly larger than that of known *Rhododendron* species. The inter‐grain areas are uniformly granular with a rough outer wall, and the ornamentation around the furrows is not prominent. This finding aligns with the conclusions of Gao et al. (2002a) and Wang et al. (2006), who noted that the majority of plants in subsect. Fortunea have smooth or nearly smooth areas around the furrows and polar regions. *R. nymphaeoides* exhibits typical characteristics of pollen from subsect. Fortunea, which indirectly supports the rationality of its systematic classification from a palynological perspective. Furthermore, according to the comparative study of pollen morphology by Zhou et al. (2008) on different pollination methods, *R. nymphaeoides* shows similarities in both pollen diameter and surface ornamentation characteristics with pollen traits associated with insect‐mediated pollination. This establishes a palynological foundation for further research on the pollination biology of *R. nymphaeoides*.

Pollen viability is fundamental for the sexual reproduction of plants and directly affects the efficiency of breeding (Li et al. 2025). The stamens and pistils of *R. nymphaeoides* mature at different times, with observations indicating that the stamens mature first. The pollen viability peaks at 11:00 a.m. on the day of flowering (91.86%), which is lower than that of *Rhododendron sinofalconeri* (with a maximum pollen viability of 92.80%) (X. Zhang 2020) and *R. longipedicellatum* (with a maximum pollen viability of 92.18%) (Li et al. 2018), but higher than that of *R. siderophyllum* (with a maximum pollen viability of 83.95%) (Bai et al. 2014) and *R. excellens* (with a maximum pollen viability of 84.43%) (Tian et al. 2024). Therefore, for genetic breeding, collecting pollen for artificial pollination experiments at 11:00 a.m. on the day of flowering may be the most advantageous. The pollen viability of *R. nymphaeoides* remains above 50% for 6 days, and during the flowering period, the pollen viability is consistently above 40%. This suggests that *R. nymphaeoides* has mechanisms to produce a large amount of pollen and effectively maintain pollen viability to ensure successful pollination. Even during the wilting stage, the pollen viability of *R. nymphaeoides* can still be maintained at 36.67%, which may be attributed to the gradual maturation and accumulation of immature pollen from the peak flowering period to the wilting stage.

The genus *Rhododendron* comprises a wide variety of species, and there are significant differences in the requirements for sucrose, boron ions, and calcium ions during the in vitro pollen culture process, as well as in the optimal culture combinations (Zhang and Geng 2012). This study indicates that the suitable solid medium for the germination of fresh pollen from *R. nymphaeoides* is 10 g·L^−1^ agar + 100 g·L^−1^ sucrose + 200 mg·L^−1^ H_3_BO_3_ + 50 mg·L^−1^ CaCl_2_. Similar to *Rhododendron* × *pulchrum* (Li et al. 2012), *Rhododendron ciliatum*, and *Rhododendron hybridum* (Zhang and Geng 2012), sucrose plays a dominant role in the pollen germination of *R. nymphaeoides*. Appropriate sucrose concentrations can significantly promote pollen germination, while concentrations that are too low or too high can lead to decreased germination rates or ruptured pollen tube tips. This may be because the suitable concentration of sucrose provides the carbon nutrients required for germination and maintains osmotic balance between the pollen and the medium (Pierson et al. 1995). Conversely, excessively high concentrations can create a hyperosmotic environment, leading to plasmolysis, which inhibits pollen germination and growth (Bo et al. 2011). Boron is an essential element for pollen germination, as it can form complexes with sucrose, facilitating transport within tissues and participating in the synthesis of pectic substances in pollen tubes (Fernando et al. 2005). Generally, the content of H_3_BO_3_ in pollen cells is low, and normal germination requires the absorption of boron from the stigma and style; therefore, an appropriate amount of H_3_BO_3_ needs to be added in in vitro germination experiments to promote effective pollen germination (Zuo et al. 2007). The results of the orthogonal experiment indicate that the influence of H_3_BO_3_ on the pollen germination of *R. nymphaeoides* is less significant than that of sucrose and CaCl_2_, but the addition of a certain amount of H_3_BO_3_ has a good promoting effect. Research by Yao and Zhao (2004) on the pollen of *Torenia fournieri* and by Zhang and Geng (2012) on the pollen characteristics of *Rhododendron ovatum* indicates that germination does not require the presence of exogenous Ca^2+^. In this study, the addition of CaCl_2_ also significantly inhibited the germination of *R. nymphaeoides* pollen, which may be due to the fact that the addition of sucrose and H_3_BO_3_ reduced the pollen's requirement for Ca^2+^, as there is already a relatively high level of free calcium within the pollen cells, sufficient to meet the needs for pollen germination and pollen tube elongation.

The storage lifespan of pollen is crucial for the preservation of germplasm resources and for addressing issues related to distant hybridization, while the storage temperature directly affects the duration of pollen storage periods (Lu et al. 2016). This study preliminarily explored the effects of different storage temperatures on the pollen viability of *R. nymphaeoides*. The results indicated that the time for which pollen can be stored at room temperature is relatively short, while low temperatures significantly extend the storage duration of pollen. This may be due to the low‐temperature, dry storage environment reducing the respiration intensity and enzyme activity of pollen cells, thereby decreasing the consumption of soluble sugars and organic acids. The viability of *R. nymphaeoides* pollen can only be maintained for about 10 days at room temperature, and the viability throughout the entire storage period is significantly lower than the corresponding measurements taken at different days after flowering. This may be because, during the flowering period, pollen continuously matures and accumulates within the anthers, and floral organs such as filaments and pedicels provide certain nutrients to the pollen, thus delaying its decline. Under low‐temperature conditions of 4°C, −20°C, and −80°C, the pollen can be stored for approximately 36, 48, and 54 days, respectively. Its viability levels are higher than those of *Rhododendron simsii* (Zuo et al. 2007), *R. ovatum* (Zhang and Geng 2012), and *Rhododendron* × *pulchrum* (Zuo et al. 2007), but lower than those of *Rhododendron wardii* (Lu et al. 2016), which can be stored for 30, 60, and 120 days at 20°C, 4°C, and −16°C, respectively. Therefore, it can be seen that *R. nymphaeoides* pollen can be stored for a longer time at −80°C, which is beneficial for addressing issues related to mismatched flowering periods with another parent in hybrid breeding and distant hybridization.

### 4.3 Breeding System and Pollination Methods of *R. nymphaeoides*

According to the classification criteria established by Dafni and Maués (1998) and Cruden (1977), the breeding system type of *R. nymphaeoides* is identified as a facultative cross‐fertilization mixed mating system, with a natural fruit set rate of 43.33%. However, very few seedlings of *R. nymphaeoides* have been found in wild populations, indicating the presence of pollination limitation (PL = 0.33) and reproductive barriers (RA = 0.15). This suggests that the species faces obstacles to natural regeneration, which may be related to site conditions such as shallow soil layers and high canopy closure that hinder seed germination. Research by Free (1993) and Reader (1977) has shown that most plants in the Ericaceae family exhibit self‐compatibility, and the self‐pollination fruit set rate of *R. nymphaeoides* is 76.67%, indicating that it can set fruit through self‐pollination. Field surveys revealed that from the third to the seventh day of flowering, the anthers of *R. nymphaeoides* gradually shed and the pollen appeared linear. By the 8th to 10th day of flowering, the corolla began to wither and droop, and the receptacle started to separate from the base, causing the petals and stamens to shift towards the style. Pollen adhered to the style, resulting in partial self‐pollination, thus indicating that *R. nymphaeoides* possesses a certain degree of self‐compatibility, consistent with the breeding systems of *R. maxiongense* (Yi et al. 2018), *R. siderophyllum* (Bai et al. 2014), and *R. excellens* (Tian et al. 2024). This study infers that the breeding system of *R. nymphaeoides* is likely evolving from self‐fertilization to cross‐fertilization, as *R. nymphaeoides*, like most rhododendrons such as *R. longipedicellatum* (Li et al. 2018), *Rhododendron aureum* (Kudo 1993), and *R. hemsleyanum* (Xie et al. 2023), has evolved the characteristic of a curved, suspended style. This spatial separation of stamens and pistils helps avoid self‐pollination. *R. nymphaeoides* is distributed along steep cliffs and in valleys, where interspecific distances are considerable and pollinators are relatively scarce, limiting cross‐pollination. The partial self‐compatibility may be a compensatory adaptation to community characteristics. Frequent distant and close crosses can lead to a decline in the fitness of its breeding system; therefore, the most suitable mating strategy is to achieve moderate hybridization (Basnett et al. 2019). The spatial separation of the anthers and stigma in *R. nymphaeoides* will significantly reduce self‐pollination, prevent inbreeding depression, and enhance the adaptability of the offspring. This study posits that *R. nymphaeoides* exhibits partial self‐compatibility, which aligns with the viewpoint of Lee et al. (2004) that species originating from smaller founding populations often experience inbreeding phenomena due to narrow distribution ranges, habitat fragmentation, and small population sizes. Self‐pollinating species tend to have weaker environmental adaptability and potential for forming new species, making self‐pollination unsuitable as a long‐term reproductive strategy (He and Liu 2003). When selecting *R. nymphaeoides* as a maternal parent, its stigma receptivity is strongest on the sixth and seventh days after flowering. At this time, artificial cross‐pollination can increase the probability of cross‐fertilization under natural conditions, promoting gene exchange between different populations.

### 4.4 Pollinating Insects of *R. nymphaeoides*

Pollination is an important process in the sexual reproduction of flowering plants. Due to their fixed growth, flowering plants require certain pollinators to facilitate pollen transfer, which mainly includes animals, wind, and water (D. Y. Zhang 2004). Research indicates that approximately 87.5% of flowering plants on Earth rely on animals for pollen transfer (Ollerton et al. 2011). Previous reports have identified effective pollinators for *R. excellens* (Tian et al. 2024), *R. longipedicellatum* (Li et al. 2018), and *Rhododendron faberi* subsp. *Prattii* (Yu et al. 2021) as the *A. cerana* and *A. dorsata* . This study found that butterfly species such as *P. paris* , *P. bianor*, *P. polytes*, and *P. protenor* are effective pollinators of *R. nymphaeoides*. Swallowtail butterflies are large and have long proboscises, which can reach the base of the corolla tube when visiting *R. nymphaeoides* flowers, allowing their abdomens or wings to contact the pollen or stigma. This study also considers *A. cerana* , *V. koreensis* , *V. velutina* , *X. appendiculata* , and *X. tranquebarorum* as effective pollinators of *R. nymphaeoides*, with *A. cerana* showing a preference for early‐opening flowers, which have strong pollen viability and high pollen output, ensuring timely pollination. The visitation frequency of bees insects may be related to their heat tolerance, cold resistance, stress resistance, and pollen transfer efficiency (Rader et al. 2013). Additionally, this study also identified small insects such as *B. eximius* , *A. plumipes* , *S. torvus* , *H. eristaloidea*, *E. arbustorum* , and *N. pellucida* as nectar robbers. In fact, nectar robbing occurs in nearly all plant species with tubular flowers. Local climatic conditions, morphological mismatches between plants and animals, resource competition and territorial disputes among insects, and foraging behaviors may collectively contribute to the occurrence of nectar robbing. These nectar robbers exhibited significantly shorter visitation durations per flower compared with the time required for effective pollination. Shorter single‐flower visitation durations correlated with higher visitation frequencies, allowing robbers to exploit more nectar resources. While nectar robbing reduces floral nectar availability, forcing pollinators to forage over longer distances, it may inadvertently promote outcrossing by facilitating pollen transfer across broader spatial scales (Basnett et al. 2019). However, studies by Xiao et al. (2022) suggest that pollinator body size and its morphological compatibility with floral structures, as well as foraging behavior, critically determine whether an insect acts as a pollinator or a robber. Specifically, the adaptive match between insect morphology and floral traits is a key factor. Based on this, we hypothesize that *B. eximius* , with its larger body size, may drive evolutionary adaptations in the floral traits of *R. nymphaeoides* through nectar robbing. Conversely, smaller insects such as *A. plumipes* , *S. torvus* , *H. eristaloidea*, *E. arbustorum* , and *N. pellucida* likely fail to enhance outcrossing due to their limited capacity to transfer pollen effectively. Additionally, *O. taurus* was identified as a pollen thief. Its pollen‐feeding behavior may reduce pollen quantity, and such declines in pollen availability or quality could impose pollination limitations on *R. nymphaeoides* (Xiao et al. 2022). Therefore, while expanding the population of *R. nymphaeoides* through artificial breeding methods, it is essential to release effective visiting insects during the flowering period to comprehensively improve the cross‐fertilization rate of *R. nymphaeoides* under natural conditions. As this study only investigated the type locality of *R. nymphaeoides*, the Hutou Mountain Scenic Area in Gulin County, Sichuan Province, due to the limitations of local weather conditions, geographical location, and insect species, it may affect factors such as flowering time, composition of pollinator species, and visit frequency. Therefore, we will further conduct pollination biology research in other regions in the subsequent studies.

## 5 Conclusion

This study divided the flowering process of *R. nymphaeoides* into five periods and systematically investigated the floral traits, pollen morphology and viability, optimal solid media for in vitro pollen germination, storage temperature screening, pollen–ovule ratio and hybridization index, stigma receptivity, the effects of different pollination methods on fruit set rates, and visiting insects. This research aimed to understand the floral syndrome and breeding system of this endangered species. The results indicate that the breeding system of *R. nymphaeoides* is a facultative cross‐fertilization mixed mating system, with floral characteristics that demonstrate adaptations for insect‐mediated pollination. At both the individual and population levels, *R. nymphaeoides* exhibits high flowering synchrony, attracting a greater number of visitors and resulting in higher fruit set rates. The spatial separation of stamens and pistils can, to some extent, avoid self‐pollination. Additionally, the morphological characteristics of *R. nymphaeoides* pollen support the rationality of its systematic classification from a palynological perspective. Its pollen viability and stigma receptivity are strong, making it suitable as a paternal parent for hybrid breeding research. The study also determined the optimal pollen collection period, the most suitable culture medium, and the best storage temperature, laying a theoretical foundation for addressing potential issues such as mismatched flowering periods and distant hybridization when using *R. nymphaeoides* as a hybrid parent in future breeding efforts. Furthermore, as *R. nymphaeoides* is found in a tourist development area with significant human disturbance, its population can be expanded through artificial breeding methods, and effective pollinators such as *P. paris* can be released during the flowering period to increase the probability of cross‐fertilization under natural conditions.

## Author Contributions

**Jun Luo:** conceptualization (equal), data curation (equal), funding acquisition (equal), investigation (equal), methodology (equal), visualization (equal), writing – original draft (equal), writing – review and editing (equal). **Meng Chen:** validation (equal), visualization (equal). **Haiyan Long:** investigation (equal), visualization (equal). **Yuting Zhu:** investigation (equal), visualization (equal). **Congjun Yuan:** supervision (equal), writing – review and editing (equal). **Kai Hu:** investigation (equal), visualization (equal). **Jin Chen:** validation (equal), visualization (equal). **Run Liu:** validation (equal), visualization (equal). **Xiaoyong Dai:** formal analysis (equal), investigation (equal), resources (equal), writing – review and editing (equal). **Fangjun Ding:** supervision (equal), writing – review and editing (equal).

## Funding

This work was funded by The Guizhou Provincial Science and Technology Foundation Project (Guizhou Science and Technology Foundation—ZK [2024] General 621), The Guizhou province forestry research project (Basic of Guizhou forestry J [2025]No.10), and The Guizhou Academy of Forestry research project (Basic of Guizhou academy of forestry [2024]No.6).

## Conflicts of Interest

The authors declare no conflicts of interest.

## Supporting information


**Table S1:** Raw data of floral syndrome and breeding system of *Rhododendron nymphaeoides*.

## Data Availability

The data that support the findings of this study are available in the Table S1 of this article.

## References

[ece372812-bib-0001] Anderson, B. , J. J. Midgley , and B. A. Stewart . 2003. “Facilitated Selfing Offers Reproductive Assurance: A Mutualism Between a Hemipteran and Carnivorous Plant.” American Journal of Botany 90, no. 7: 1009–1015. 10.3732/ajb.90.7.1009. 21659199

[ece372812-bib-0002] Bai, T. , W. L. Guan , J. Song , W. J. Xie , and S. F. Li . 2014. “Flowering Characteristics and Breeding System of *Rhododendron siderophyllum* .” Journal of West China Forestry Science 43, no. 1: 47–53. 10.16473/j.cnki.xblykx1972.2014.01.017.

[ece372812-bib-0003] Barrett, S. C. H. 2010. “Understanding Plant Reproductive Diversity.” Philosophical Transactions of the Royal Society, B: Biological Sciences 365: 99–109. 10.1098/rstb.2009.0199. PMC2842705 20008389

[ece372812-bib-0004] Barrett, S. C. H. , and L. D. Harder . 1996. “Ecology and Evolution of Plant Mating.” Trends in Ecology & Evolution 11, no. 2: 73–79. 10.1016/0169-5347(96)81046-9. 21237765

[ece372812-bib-0005] Basnett, S. , R. Ganesan , and S. M. Devy . 2019. “Floral Traits Determine Pollinator Visitation in *Rhododendron* Species Across an Elevation Gradient in the Sikkim Himalaya.” Alpine Botany 129, no. 2: 81–94. 10.1007/s00035-019-00225-3.

[ece372812-bib-0006] Bhardwaj, M. , and C. G. Eckert . 2001. “Functional Analysis of Synchronous Dichogamy in Flowering Rush, *Butomus umbellatus* (Butomaceae).” American Journal of Botany 88, no. 12: 2204–2213. 10.2307/3558382. 21669653

[ece372812-bib-0007] Bie, P. F. , T. Tang , J. Y. Hu , and W. Jiang . 2018. “Flowering Phenology and Breeding System of an Endangered and Rare Species *Urophysa rockii* (Ranunculaceae).” Acta Ecologica Sinica 38, no. 11: 3899–3908. 10.5846/stxb201705160905.

[ece372812-bib-0008] Bo, Z. G. , S. H. Du , and X. M. Zhang . 2011. “Study on the Flowering Habit and Pollen Viability of *R. arborescens* Planch.” Journal of Anhui Agricultural Sciences 39, no. 8: 4562–4563. 10.13989/j.cnki.0517-6611.2011.08.225.

[ece372812-bib-0009] Cao, M. H. , Q. Y. Zhao , C. M. Wei , H. Q. Huang , and M. J. Huang . 2022. “Study on the Flowering Traits and Breeding Systems of Three Impatiens Species.” Plant Science Journal 40, no. 3: 291–301. 10.11913/PSJ.2095-0837.2022.30291.

[ece372812-bib-0010] Cruden, R. W. 1977. “Pollen‐Ovule Ratios: A Conservative Indicator of Breeding Systems in Flowering Plants.” Evolution 31, no. 1: 32–46. 10.2307/2407542. 28567723

[ece372812-bib-0011] Dafni, A. 1992. Pollination Ecology: A Practical Approach. Oxford University Press.

[ece372812-bib-0012] Dafni, A. , and M. M. Maués . 1998. “A Rapid and Simple Procedure to Determine Stigma Receptivity.” Sexual Plant Reproduction 11, no. 3: 177–180. 10.1007/s004970050138.

[ece372812-bib-0013] Dai, G. L. , K. Qin , Y. L. Cao , E. N. Jiao , and B. Zhang . 2013. “Characteristics of Floral Dynamic and Breeding System of *Lyciun ruthenicum* .” Guihaia 33, no. 1: 126–132. 10.3969/j.issn.1000-3142.2013.01.023.

[ece372812-bib-0014] Dai, P. F. , and D. Y. Tan . 2011. “Floral Biological Characteristics of *Saussurea involucrate* in Relation to Ecological Adaptation.” Chinese Journal of Plant Ecology 35, no. 1: 56–65. 10.3724/SP.J.1258.2011.00056.

[ece372812-bib-0015] Dai, X. Y. , L. X. Deng , Y. P. Ma , and C. H. Yang . 2022. Rhododendron of Guizhou, China. Guizhou Science and Technology Press.

[ece372812-bib-0016] Dellinger, A. S. 2020. “Pollination Syndromes in the 21st Century: Where Do We Stand and Where May We Go?” New Phytologist 228, no. 4: 1193–1213. 10.1111/nph.16793. 33460152

[ece372812-bib-0017] Devaux, C. , C. Lepers , and E. Porcher . 2014. “Constraints Imposed by Pollinator Behaviour on the Ecology and Evolution of Plant Mating Systems.” Journal of Evolutionary Biology 27, no. 7: 1413–1430. 10.1111/jeb.12380. 24750302

[ece372812-bib-0018] Escaravage, N. , and J. Wagner . 2004. “Pollination Effectiveness and Pollen Dispersal in a *Rhododendron ferrugineum* (Ericaceae) Population.” Plant Biology 6, no. 5: 606–615. 10.1055/s-2004-821143. 15375732

[ece372812-bib-0019] Fernando, D. D. , M. D. Lazzaro , and J. N. Owens . 2005. “Growth and Development of Conifer Pollen Tubes.” Sexual Plant Reproduction 18, no. 4: 149–162. 10.1007/s00497-005-0008-y.

[ece372812-bib-0020] Free, J. B. 1993. Insect Pollination of Crops. Academic Press.

[ece372812-bib-0021] Gao, L. M. , C. Q. Zhang , D. Z. Li , and Z. X. Wei . 2002a. “Pollen Morphology of the Rhodoreae (Ericaceae) and Its Systematic Implication.” Acta Botanica Yunnanica 24, no. 4: 471–482.

[ece372812-bib-0022] Gao, L. M. , C. Q. Zhang , D. Z. Li , and Z. X. Wei . 2002b. “Pollen Morphology of *Rhododendron* Subgenus *Azaleastrum* .” Journal of Wuhan Botanical Research 20, no. 3: 177–181.

[ece372812-bib-0023] Goodwillie, C. , R. D. Sargent , C. G. Eckert , et al. 2010. “Correlated Evolution of Mating System and Floral Display Traits in Flowering Plants and Its Implications for the Distribution of Mating System Variation.” New Phytologist 185: 311–321. 10.1111/j.1469-8137.2009.03043.x. 19807872

[ece372812-bib-0024] Hao, Z. Y. , J. Song , Y. F. Li , and W. L. Guan . 2025. “Determination of Pollen Viability of Different Varieties of Encore Azalea and Analysis of Hybridization Compatibility.” Journal of Central South University of Forestry & Technology 45, no. 4: 76–86. 10.14067/j.cnki.1673-923x.2025.04.007.

[ece372812-bib-0025] He, Y. P. , and J. Q. Liu . 2003. “A Review on Recent Advances in the Studies of Plant Breeding System.” Acta Pharmacologica Sinica 27, no. 2: 151–163.

[ece372812-bib-0026] Herrera, C. M. 1986. “Vertebrate‐Dispersed Plants: Why They Don't Behave the Way They Should.” Frugivores and Seed Dispersal 15: 5–18. 10.1007/978-94-009-4812-9_2.

[ece372812-bib-0027] Jain, A. , M. K. Pandit , S. Elahi , A. Jain , A. Bhaskar , and V. Kumar . 2000. “Reproductive Behaviour and Genetic Variability in Geographically Isolated Populations of *Rhododendron arboreum* (Ericaceae).” Current Science 79, no. 9: 1377–1381. http://www.jstor.org/stable/24105291.

[ece372812-bib-0028] Ji, S. , S. J. Yan , L. Yang , J. W. Ye , and J. W. Liao . 2023. “Flower Visiting Insects Species and Behaviors in *Bidens pilosa* .” Journal of Northeast Forestry University 51, no. 2: 77–81. 10.13759/j.cnki.dlxb.2023.02.018.

[ece372812-bib-0029] Kudo, G. 1993. “Relationship Between Flowering Time and Fruit Set of the Entomophilous Alpine Shrub, *Rhododendron aureum* (Ericaceae), Inhabiting Snow Patches.” American Journal of Botany 80, no. 11: 1300–1304. 10.1002/j.1537-2197.1993.tb15368.x.

[ece372812-bib-0030] Lander, T. A. , S. A. Haris , P. J. Cremona , and D. H. Boshier . 2019. “Impact of Habitat Loss and Fragmentation on Reproduction, Dispersal and Species Persistence for an Endangered Chilean Tree.” Conservation Genetics 20, no. 5: 973–985. 10.1007/s10592-019-01187-z.

[ece372812-bib-0031] Lee, P. L. M. , R. M. Patel , R. S. Conlan , S. J. Wainwright , and C. R. Hipkin . 2004. “Comparison of Genetic Diversities in Native and Alien Populations of Hoary Mustard ( *Hirschfeldia incana* [L.] Lagreze‐Fossat).” International Journal of Plant Sciences 165, no. 5: 833–843. 10.1086/422043.

[ece372812-bib-0032] Li, N. , X. B. Yang , X. H. Wang , S. X. Meng , and S. B. Peng . 2025. “Pollen Viability and Storage Characteristics of *Betula albosinensis* .” Journal of Northwest Forestry University 40, no. 2: 64–72. 10.3969/j.issn.1001-7461.2025.02.07.

[ece372812-bib-0033] Li, T. Q. , X. F. Liu , Z. H. Li , et al. 2018. “Study on Reproductive Biology of *Rhododendron longipedicellatum*: A Newly Discovered and Special Threatened Plant Surviving in Limestone Habitat in Southeast Yunnan, China.” Frontiers in Plant Science 9: 33. 10.3389/fpls.2018.00033. 29445383 PMC5797782

[ece372812-bib-0034] Li, Y. P. , K. Chen , Y. Q. Wang , and F. X. Luo . 2012. “The Test of Germination of Azalea Pollen in Culture Solution and Its Storage Methods.” Journal of Jinling Institute of Technology 28, no. 3: 56–61. 10.16515/j.cnki.32-1722/n.2012.03.017.

[ece372812-bib-0035] Liu, D. T. , G. Yao , Y. R. Cao , P. G. Zhu , and Y. P. Ma . 2025. “Floral Syndrome and Breeding System of *Rhododendron griersonianum* .” Acta Ecologica Sinica 45, no. 2: 889–897. 10.20103/j.stxb.202405131084.

[ece372812-bib-0036] Liu, D. X. , Q. F. Wang , and C. F. Yang . 2022. “Flower Diversity and Pollination Strategy in Araceae.” Biodiversity Science 30, no. 3: 21426. 10.17520/biods.2021426.

[ece372812-bib-0037] Liu, L. D. , L. Chen , L. Zhang , C. L. Li , and Y. B. Gao . 2004. “Flowering Characteristics and Pollination Ecology of *Scabiosa tschiliensis* .” Acta Ecologica Sinica 24, no. 4: 718–723. 10.3321/j.issn:1000-0933.2004.04.010.

[ece372812-bib-0038] Liu, Q. , J. Yang , X. R. Wang , and Y. Zhao . 2023. “Studies on Pollen Morphology, Pollen Vitality and Preservation Methods of *Gleditsia sinensis* Lam. (Fabaceae).” Forests 14: 243. 10.3390/f14020243.

[ece372812-bib-0039] Loveless, M. D. , and J. L. Hamrick . 1984. “Ecological Determinants of Genetic Structure in Plant Populations.” Annual Review of Ecology and Systematics 15: 65–95. http://www.jstor.org/stable/2096943.

[ece372812-bib-0040] Lu, L. , L. C. Peng , J. Song , S. F. Li , W. J. Xie , and S. F. Li . 2016. “Study on Viability and Storage Capacity of Pollen Among Different *Rhododendron lapponicum* Cultivars.” Journal of Shanxi Agricultural Sciences 44, no. 2: 175–178. 10.3969/j.issn.1002-2481.2016.02.11.

[ece372812-bib-0041] Luo, J. , X. Y. Dai , J. Chen , S. He , C. J. Yuan , and D. L. Luo . 2025. “Study on the Characteristics of Genetic Diversity and Population Structure of a Rare and Endangered Species of *Rhododendron nymphaeoides* (Ericaceae) Based on Microsatellite Markers.” BMC Plant Biology 25: 310. 10.1186/s12870-025-06362-8. 40069601 PMC11895177

[ece372812-bib-0042] Luo, J. , X. Y. Dai , J. Chen , C. J. Yuan , H. D. Wang , and D. L. Luo . 2025. “Community Characteristics and Population Dynamics of an Endangered *Rhododendron nymphaeoides* .” Ecology and Evolution 15: e71268. 10.1002/ece3.71268. 40225893 PMC11985896

[ece372812-bib-0043] Mao, Z. J. , Y. F. Yang , and L. J. Hou . 2000. “A Study on the Pollen Morphology of Rhododendron in Northeast of China.” Bulletin of Botanical Research 20, no. 1: 58–62.

[ece372812-bib-0044] Marbaniang, E. J. , N. Venugopal , S. Vema , R. Raina , A. Khajuria , and K. Gautam . 2018. “Floral Biology and Embryological Studies Are Important for Conservation of Threatened Plants Having Reproductive Bottlenecks: A Case Study of *Ilicum grifithiü* Hook. F. & Thomson.” Current Science 114, no. 3: 576. http://www.jstor.org/stable/26495111.

[ece372812-bib-0045] Mcintosh, M. E. 2002. “Flowering Phenology and Reproductive Output in Two Sister Species of *Ferocactus* (Cactacear).” Plant Ecology 159, no. 1: 1–13. 10.1023/A:1015589002987.

[ece372812-bib-0046] Nicotra, A. B. , O. K. Atkin , S. P. Bonser , et al. 2010. “Plant Phenotypic Plasticity in a Changing Climate.” Trends in Plant Science 15: 684–692. 10.1016/j.tplants.2010.09.008. 20970368

[ece372812-bib-0047] Niu, L. J. , Y. Y. Hou , S. J. Wen , et al. 2025. “Floral Nectar: Secretion Behavior, Cost and Regulation.” Biodiversity Science 33, no. 8: 25142. 10.17520/biods.2025142.

[ece372812-bib-0048] Ollerton, J. , R. Winfree , and S. Tarrant . 2011. “How Many Flowering Plants Are Pollinated by Animals?” Oikos 120: 321–326. 10.1111/j.1600-0706.2010.18644.x.

[ece372812-bib-0049] Pierson, E. S. , Y. Q. Li , H. Q. Zhang , M. T. M. Willemse , H. F. Linskens , and M. Cresti . 1995. “Pulsatory Growth of Pollen Tubes: Investigation of a Possible Relationship With the Periodic Distribution of Cell Wall Components.” Acta Botanica Neerlandica 44, no. 2: 121–128.

[ece372812-bib-0050] Rader, R. , W. Edwards , D. A. Westcott , S. A. Cunningham , and B. G. Howlett . 2013. “Diurnal Effectiveness of Pollination by Bees and Flies in Agricultural *Brassica rapa* : Implications for Ecosystem Resilience.” Basic and Applied Ecology 14, no. 1: 20–27. 10.1016/j.baae.2012.10.011.

[ece372812-bib-0051] Reader, R. J. 1977. “Bog Ericad Flowers: Self‐Compatibility and Relative Attractiveness to Bees.” Canadian Journal of Botany 55, no. 17: 2279–2287. 10.1139/b77-259.

[ece372812-bib-0052] Spigler, R. B. , and S. Kalisz . 2013. “Phenotypic Plasticity in Mating System Traits in the Annual *Collinsia verna* .” Botany 91: 597–604. 10.1139/cjb-2012-0227.

[ece372812-bib-0053] Stout, J. C. 2007. “Pollination of Invasive *Rhododendron ponticum* (Ericaceae) in Ireland.” Apidologie 38, no. 2: 198–206. 10.1051/apido:2006071.

[ece372812-bib-0054] Sun, J. F. , Y. B. Gong , S. S. Renner , S. Q. Huang , and H. M. Wilbur . 2008. “Multifunctional Bracts in the Dove Tree *Davidia involucrata* (Nyssaceae: Cornales): Rain Protection and Pollinator Attraction.” American Naturalist 171, no. 1: 119. 10.1086/523953. 18171156

[ece372812-bib-0055] Swift, J. F. , S. A. Smith , E. S. Menges , B. Basstiner , and C. E. Edwards . 2016. “Analysis of Mating System and Genetic Structure in the Endangered, Amphicarpic Plant, Lewton's Polygala ( *Polygala lewtonii* ).” Conservation Genetics 17, no. 6: 1269–1284. 10.1007/s10592-016-0860-3.

[ece372812-bib-0056] Tai, C. R. , K. Zhao , Y. F. Yang , Y. Wu , W. Zhai , and Y. W. Tong . 2023. “Flowering Biological Characteristics and Breeding System of the Rare and Endangered Plant of *Sinojackia microcarpa* .” Bulletin of Botanical Research 43, no. 2: 311–320. 10.7525/j.issn.1673-5102.2023.02.016.

[ece372812-bib-0057] Tamura, S. , and G. Kudo . 2000. “Wind Pollination and Insect Pollination of Two Temperate Willow Species, *Salix miyabeana* and *Salix sachalinensis* .” Plant Ecology 147, no. 2: 185–192. 10.1023/A:1009870521175.

[ece372812-bib-0058] Tang, L. , and B. Han . 2007. “Effects of Floral Display on Pollinator Behavior and Pollen Dispersal.” Biodiversity Science 15, no. 6: 680–686. 10.1360/biodiv.070222.

[ece372812-bib-0059] Tian, X. L. , P. P. Tao , C. L. Huang , and C. Q. Zhang . 2024. “Study on Pollination Biology of the Vulnerable Plant *Rhododendron excellens* .” Acta Botanica Boreali‐Occidentalia Sinica 44, no. 11: 1820–1827. 10.7606/j.issn.1000-4025.20240304.

[ece372812-bib-0060] Wan, H. X. , H. P. Deng , P. He , Q. Q. Jiang , and Q. Liu . 2018. “Breoding System and Pollination Biology of Endangered *Plantago fengdouensis* .” Acta Eologica Sinica 38, no. 11: 4018–4026. 10.5846/stxb201707301372.

[ece372812-bib-0061] Wang, Y. G. , G. Z. Li , X. X. Qi , and Z. L. Ou . 2006. “Pollen Morphology of *Rhododendron* and Its Taxonomic Implication.” Guihaia 26, no. 2: 113–119.

[ece372812-bib-0062] Weiss, M. R. 1991. “Floral Colour Changes as Cues for Pollinators.” Nature 354, no. 6350: 227–229. 10.1038/354227a0.

[ece372812-bib-0063] Wu, Y. , X. Y. Duan , G. L. Liu , Y. Xiang , B. Shu , and Q. J. Li . 2021. “Vegetation Context Modifies Selection on Flowering Start and Plant Height in an Orchid Perennial Herb.” Journal of Plant Ecology 14, no. 5: 934–944. 10.1093/jpe/rtab048.

[ece372812-bib-0064] Wu, Y. M. , X. L. Shen , L. Tong , et al. 2022. “Reproductive Biology of an Endangered Lithophytic Shrub and Implications for Its Conservation.” BMC Plant Biology 22, no. 1: 80. 10.1186/s12870-022-03466-3. 35193519 PMC8862588

[ece372812-bib-0065] Wyatt, R. 1983. “Pollinator‐Plant Interactions and the Evolution of Breeding Systems.” In Pollination Biology, 51–95. Academic Press.

[ece372812-bib-0066] Xiao, H. W. , Y. B. Huang , Q. Wang , and Y. K. Wei . 2022. “Diversity of Visiting Insects and Changes of Pollinator Behavior in Alpine Species *Salvia castanea* Diels (Lamiaceae).” Acta Ecologica Sinica 42, no. 5: 1841–1853. 10.5846/stxb202101070071.

[ece372812-bib-0067] Xiao, J. 2010. Reasonable Assessment of Land Use Planning in Gulin. Sichuan Agricultural University.

[ece372812-bib-0068] Xie, M. , Y. P. Ma , Y. Y. Cao , D. T. Liu , Z. H. Li , and H. Ma . 2023. “Research on Floral Syndrome and Breeding Systems of *Rhododendron hemsleyanum*, a Plant Species With Extremely Small Populations.” Journal of Yunnan Agricultural University (Natural Science) 38, no. 1: 95–103. 10.12101/j.issn.1004-390X(n).202207033.

[ece372812-bib-0069] Yao, C. Y. , and J. Zhao . 2004. “Effects of Calcium and Boron on Pollen Germination and Pollen Tube Growth of *Torenia fournieri* .” Journal of Wuhan Botanical Research 22, no. 1: 1–7.

[ece372812-bib-0070] Ye, J. T. , S. Z. Mao , X. H. Hu , et al. 2022. “Flowering Biological Characteristics and Breeding System of *Ardisia humilis* .” Guihaia 43, no. 2: 377–387. 10.11931/guihaia.gxzw202204043.

[ece372812-bib-0071] Yi, C. R. , S. L. Zheng , Q. Liu , K. Gu , G. Q. Liu , and J. L. Zhang . 2018. “Preliminary Study on the Breeding System and Crossbreeding of *Rhododendron maxiongense* (Ericaceae).” Journal of Yunnan Agricultural University (Natural Science) 33, no. 1: 99–105. 10.12101/j.issn.1004-390X(n).201611062.

[ece372812-bib-0072] Yu, H. Q. , W. Shuai , X. H. Jiang , et al. 2021. “Study on the Breeding System of *Rhododendron prattii* Franch.” Journal of Sichuan Forestry Science and Technology 42, no. 5: 40–47. 10.12172/202108110001.

[ece372812-bib-0073] Yu, Y. S. , H. X. Lu , W. F. Song , Z. Y. Zhang , and X. W. Zhang . 2013. “Comparisons of Nectar Quantity and Sugar Concentration of Pomegranate Blossom in a Day.” Agricultural Science and Technology 14, no. 1: 69–71. 10.16175/j.cnki.1009-4229.2013.01.035.

[ece372812-bib-0074] Zhang, C. Y. , and X. M. Geng . 2012. “Comparative Study on Methods for Testing Pollen Viability of the Six Species From Genus *Rhododendron* .” Plant Science Journal 30, no. 1: 92–99. 10.3724/SP.J.1142.2012.10092.

[ece372812-bib-0075] Zhang, D. Y. 2004. Plant Life‐History Evolution and Reproductive Ecology, 153. Science Press.

[ece372812-bib-0076] Zhang, L. F. , and L. H. Qiu . 2017. “Flowering Phenology, Floral Traits and Breeding System of *Platycrater arguta* .” Guihaia 37, no. 10: 1301–1311. 10.11931/guihaia.gxzw201612006.

[ece372812-bib-0077] Zhang, W. , C. B. He , and Y. B. Gong . 2019. “Pollinator Attraction and Outcrossing Strategies in Iris.” Plant Science Journal 37, no. 5: 672–681. 10.11913/PSJ.2095-0837.2019.50672.

[ece372812-bib-0078] Zhang, X. 2020. Reproductive Biology and Conservation Genetics of Rhododendron sinofalconeri. Chinese Academy of Forestry.

[ece372812-bib-0079] Zhang, Z. , Y. L. Liang , M. H. Xu , C. H. Fu , Z. Q. Li , and Z. G. Meng . 2025. “Research on the Biological Characteristics of *Gentiana crassicaulis* Pollination.” Journal of Chinese Medicinal Materials 48, no. 7: 1626–1631. 10.13863/j.issn1001-4454.2025.07.004.

[ece372812-bib-0080] Zhou, L. Y. , Q. L. Liu , and L. X. Shao . 2008. “Comparison of the Pollen Morphological Characters Between Anthophilous Flowers and Anemophilous Flowers.” Journal of Shanghai Jiao Tong University (Agricultural Science) 26, no. 3: 177–182.

[ece372812-bib-0081] Zuo, D. D. , J. Ming , C. Liu , and L. N. Wang . 2007. “Advance in Technique of Plant Pollen Viability.” Journal of Anhui Agricultural Sciences 35, no. 16: 4742–4745. 10.13989/j.cnki.0517-6611.2007.16.015.

